# Design, Synthesis and Structure-activity Studies of Rhodanine Derivatives as HIV-1 Integrase Inhibitors

**DOI:** 10.3390/molecules15063958

**Published:** 2010-06-01

**Authors:** Kavya Ramkumar, Vladimir N. Yarovenko, Alexandra S. Nikitina, Igor V. Zavarzin, Mikhail M. Krayushkin, Leonid V. Kovalenko, Adrian Esqueda, Srinivas Odde, Nouri Neamati

**Affiliations:** 1 Department of Pharmacology and Pharmaceutical Sciences, University of Southern California, School of Pharmacy, 1985 Zonal Avenue, PSC 304, Los Angeles, CA 90033, USA; E-Mails: ramkumar@usc.edu (K.R.); adrianesqueda@hotmail.com (A.E.); odde@pharmacy.usc.edu (S.O.); 2 N.D. Zelinsky Institute of Organic Chemistry, Russian Academy of Sciences, Leninsky avenue, 47, 119991 Moscow, Russia; E-Mail: yarovladimir@tochka.ru (V.N.Y); 3 D.I. Mendeleev Russian University for Chemical Technology, Miusskaya sq., 9, 125047 Moscow, Russia

**Keywords:** rhodanine, HIV-1, integrase, APE1, 2-thioxo-4-thiazolidinone, docking

## Abstract

Raltegravir was the first HIV-1 integrase inhibitor that gained FDA approval for use in the treatment of HIV-1 infection. Because of the emergence of IN inhibitor-resistant viral strains, there is a need to identify innovative second-generation IN inhibitors. Previously, we identified 2-thioxo-4-thiazolidinone (rhodanine)-containing compounds as IN inhibitors. Herein, we report the design, synthesis and docking studies of a series of novel rhodanine derivatives as IN inhibitors. All these compounds were further tested against human apurinic/apyrimidinic endonuclease 1 (APE1) to determine their selectivity. Two compounds showed significant cytotoxicity in a panel of human cancer cell lines. Taken together, our results show that rhodanines are a promising class of compounds for developing drugs with antiviral and anticancer properties.

## 1. Introduction

Raltegravir (MK-0518) is the first HIV-1 integrase (IN) inhibitor approved for the treatment of HIV-1 infection in treatment-experienced patients, and heralds a new era in HIV/AIDS treatment [1,2]. IN inhibitors differ from other classes of antiretroviral agents in that they target the integration stage in the viral life cycle. The integration step, catalyzed by IN, results in the insertion of proviral DNA into the host genome. This occurs as a two-stage reaction. First, the proviral DNA is primed for integration by an endonucleolytic cleavage at its 3’ end. This step is called 3’-processing and takes place in the cytoplasm of an infected cell. IN remains bound to the processed viral DNA and translocates into nucleus aided by several interacting cellular cofactors. Inside the nucleus, IN catalyzes a staggered cleavage in the host DNA. The nucleophilic hydroxyl group at the 3’ end of the viral DNA is then inserted into the host DNA by a trans-esterification reaction. This process is called the strand transfer reaction. These two steps catalyzed by IN are critical for HIV-1 replication and infection [3,4,5]. 

Nearly two decades of intensive efforts towards developing clinically useful HIV-1 IN inhibitors has resulted in a rich database of structural information [6,7,8]. This is particularly useful in designing second generation inhibitors in order to overcome the challenges faced with emerging resistant strains against current IN inhibitors, including raltegravir. Among the various structural classes, β-diketoacid based pharmacophores have shown high potency and selectivity towards inhibition of the IN-catalyzed strand transfer [9,10]. S-1360, a β-diketoacid bioisostere, was the one of first IN targeted antiretroviral agent to undergo human clinical trials. Although it failed to show clinical efficacy, S-1360 has served as a good template for the design of several second-generation IN inhibitors [11,12,13]. 

**Figure 1:**
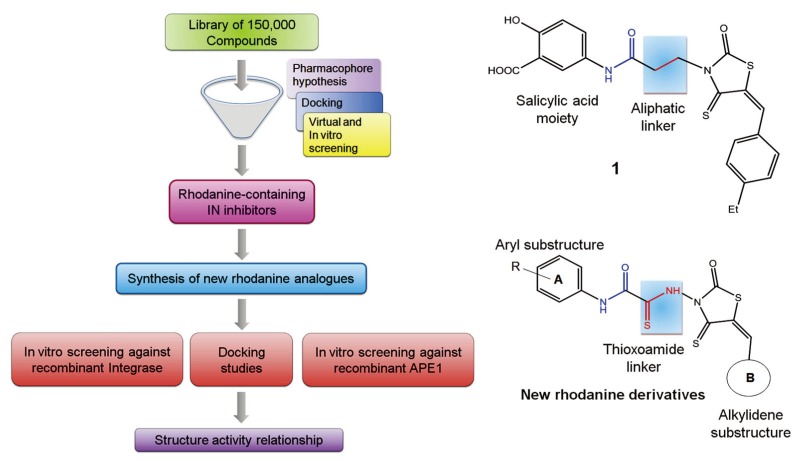
Design of new rhodanine derivatives and structural modifications for SAR study.

Previously, we identified rhodanine-containing compounds as IN inhibitors through a common feature pharmacophore search based on β−diketoacid bioisosteres (Figure 1) [14]. We reported that compounds containing both a rhodanine moiety and a salicylic acid substitution exhibited significant IN inhibition. The most potent compound **1** inhibited IN catalytic activities with IC_50_ values of 15 μM and 11 μM for 3’-processing and strand transfer, respectively. This prompted us to explore other modifications in the substructures of compound **1** (Figure 1) and study their effects on IN inhibition. Another earlier study also similarly applied a caffeic acid phenylester (CAPE)-based pharmacophore approach and identified rhodanine-containing compounds bearing a phenylsulfonamide moiety as IN inhibitors [15].

**Figure 2:**
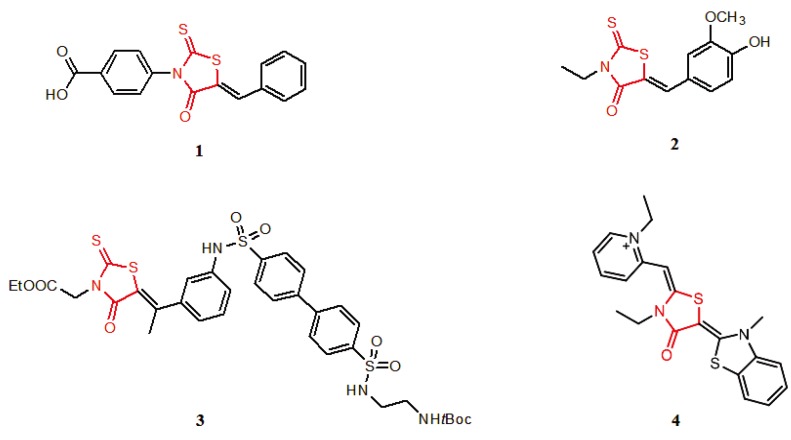
Structures of representative rhodanine derivatives with diverse biological activities [17,18,19,20]; (**2**) JSP-1 inhibitor; (**3**) PDE4 inhibitor; (**4**) HCV NS3 Protease inhibitor; (**5**) Antitumor agent (rhodanine moiety is highlighted in red).

Several rhodanine-based small molecules have also known to inhibit various enzymes such as dual-specificity phosphatases, HCV NS3 protease, phosphodiesterases, *etc*. (Figure 2) [16,17,18,19]. The central rhodanine scaffold is commonly found in several antivirals, antimicrobials and antitumor agents [20,21]. In all these structures, modifications in the alkylidene chain and in the rhodanine moiety appear to influence biological activity. While there is diverse literature on rhodanine-containing compounds as various enzyme inhibitors [22], there is limited information regarding their use as HIV-1 IN inhibitors. Herein, we report the synthesis and structure-activity relationship (SAR) for a series of rhodanine derivatives that inhibit IN in the low micromolar range. We further characterized the selectivity of these compounds for IN by counter-screening against APE1, another DNA binding enzyme, and analyzed their binding modes in the IN and APE1 active sites. Finally, we evaluated the antiproliferative effects of these rhodanines against a panel of human cancer cell lines.

## 2. Results and Discussion

### 2.1. Chemistry

**Scheme 1:**
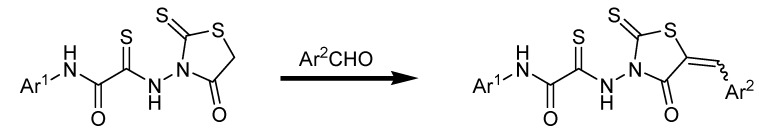
Synthesis of compounds **6**–**54**.

In our earlier studies, we identified rhodanine-containing compounds through a pharmacophore-based high-throughput screening. We found that the presence of both a rhodanine core and a salicylic acid group improved IN inhibitory effects [14]. Furthermore, substitutions in the aryl substructure and at the 4-position in the rhodanine nucleus (alkylidene substructure) also appeared to influence IN inhibition potency. Based on this, we synthesized several 2-thioxo-1,3-thiazolidin-4-ones bearing modifications in both the aryl and the alkylidene substructures to explore their effect on IN catalytic activities and understand their SAR. These rhodanines were synthesized according to previously a reported procedure (Scheme 1) [23]. All of these new analogues possessed a five-membered rhodanine moiety as a common structural unit and are linked to various substituted aromatic groups via alkylidene and four-carbon aliphatic thioxoamide linkers (Figure 1).

### 2.2. In vitro IN inhibitory activities and SAR of rhodanine derivatives

Inhibition of IN catalytic activities, 3’-processing and strand transfer, was determined using an *in vitro* enzymatic assay. Compounds with a weakly electron-withdrawing chlorophenyl group in substructure B (e.g., **6**–**8**) showed no IN inhibition at the highest concentration tested (Table 1). Interestingly, compound **9**, which has a methoxy substitution in ring A, inhibited both 3’-processing and strand transfer with an equal potency (IC_50_ value of 33 μM). Substitution with the weakly basic and bulky indole group abolished any activity (compound **10**). Compound **11**, with a stronger electron-withdrawing substitution in substructure B, had a greater potency towards IN inhibition with an IC_50_ value of 58 ± 4 μM and 20 ± 6 μM for 3’-processing and strand transfer, respectively. 

**Table 1:** Inhibition of HIV-1 IN and APE-1 catalytic activities by rhodanines **6**–**11**.

Compound	Structure	Inhibition of IN catalytic activity IC_50_ (µM)		Inhibition of APE1 catalytic activityIC_50_ (μM)
		3’-Processing	Strand Transfer	
**6**	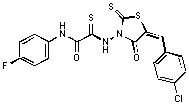	>100	>100	>100
**7**	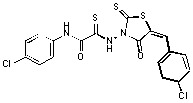	>100	>100	45 ± 51
**8**	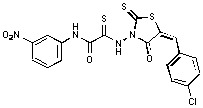	>100	>100	76 ± 5
**9 **	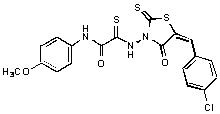	33	33	47 ± 2
**10 **	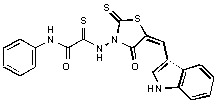	>100	>100	>100
**11 **	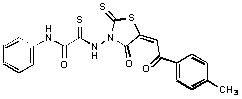	58 ± 4	20 ± 6	>100

Compounds **12**–**21** have a relatively stronger electron-withdrawing nitrofuran ring in substructure B. Again, we observed that electron-withdrawing groups in ring A led to moderate inhibitory activity, while replacement by an electron-donating group such as a methoxyl improved activity further (Table 2). 

**Table 2:** Inhibition of HIV-1 IN and APE1 catalytic activities by rhodanines **12**–**21**.

Compound	Structure	Inhibition of IN catalytic activity IC_50_ (µM)		Inhibition of APE1 catalytic activity IC_50_ (μM)
		3’-Processing	Strand Transfer	
**12**	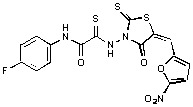	100	93	89 ± 13
**13**	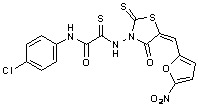	68 ± 46	41 ± 27	93
**14**	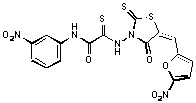	58	58	>100
**15**	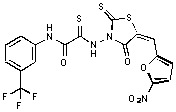	61 ± 35	56 ± 11	-
**16**	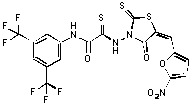	>100	74 ± 20	>100
**17**	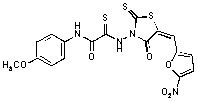	77 ± 10	60	>100
**18**	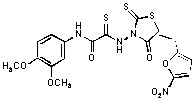	34 ± 10	14 ± 5	>100
**19**	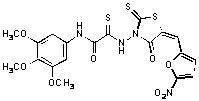	40 ± 28	25 ± 11	>100
**20**	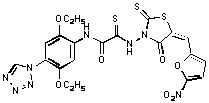	>100	85 ± 21	>100
**21**	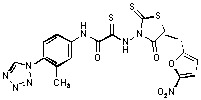	>100	>100	>100

The presence of a basic moiety such as tetrazole ring at position 4 in ring A led to a loss of activity. Interestingly, introduction of electron-donating group in substructure B did not seem favorable for IN enzyme binding, as all of the compounds **22**–**26** were inactive (Table 3). 

**Table 3:** Inhibition of HIV-1 IN and APE1 catalytic activities by rhodanines **22**–**26**.

Compound	Structure	Inhibition of IN catalytic activity IC_50_ (µM)		Inhibition of APE1 catalytic activityIC_50_ (μM)
		3’-Processing	Strand Transfer	
**22**	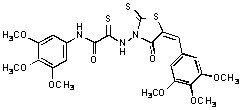	>100	90	>100
**23**	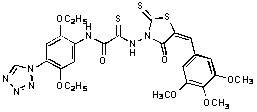	>100	>100	83
**24**	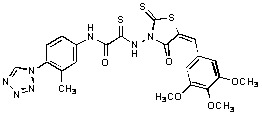	>100	>100	>100
**25**	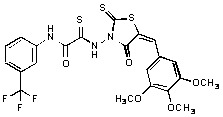	>100	>100	>100
**26**	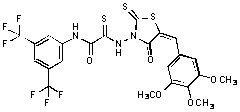	>100	>100	-

Since the presence of an electron-donating methoxy group in ring A seemed important for IN inhibitory activity, we synthesized analogs with trimethoxy substitution on ring A (Table 4). Substructure B was also modified using various substituted phenols. Electron-withdrawing substituents like Cl, Br and NO_2_ led to increased activity. Compound **35** with a salicylic acid had only a moderate effect on IN inhibition, while compound **34** with a 2-phenyldiazaphenol moiety inhibited 3’-processing and strand transfer, with IC_50_ values of 33 ± 19 μM and 26 ± 14, respectively. Aliphatic and basic substitutions had a negative effect on activity. 

**Table 4:** Inhibition of HIV-1 IN and APE1 catalytic activities by rhodanines **27**–**40**.

Compound	Structure	Inhibition of IN catalytic activity IC_50_ (µM)		Inhibition of APE1 catalytic activityIC_50_ (μM)
		3’-Processing	Strand Transfer	
**27**	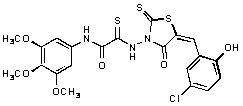	83 ± 25	20	>100
**28**	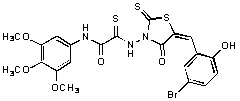	44 ± 13	30 ± 19	>100
**29**	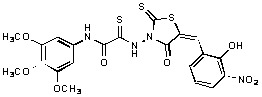	74 ± 18	72 ± 23	>100
**30**	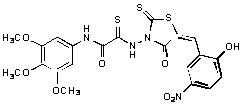	47 ± 30	>100	>100
**31**	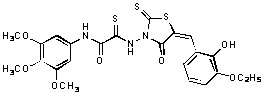	>100	>100	>100
**32**	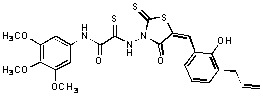	82 ± 5	83 ± 3	>100
**33**	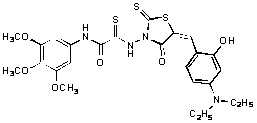	>100	>100	>100
**34**	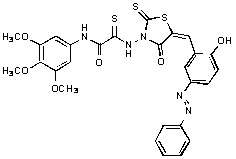	33 ± 19	26 ± 14	>100
**35**	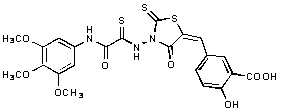	45 ± 24	61 ± 11	>100
**36**	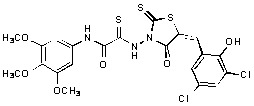	46 ± 5	>100	>100
**37**	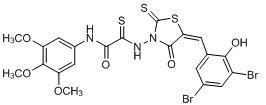	33 ± 7	35 ± 6	>100
**38**	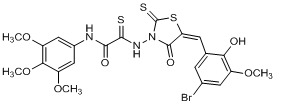	51 ± 25	32 ± 11	65 ± 33
**39**	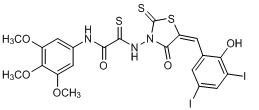	47 ± 20	31 ± 16	>100
**40**	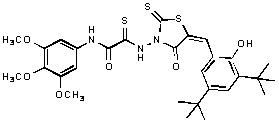	>100	95	>100

**Figure 3:**
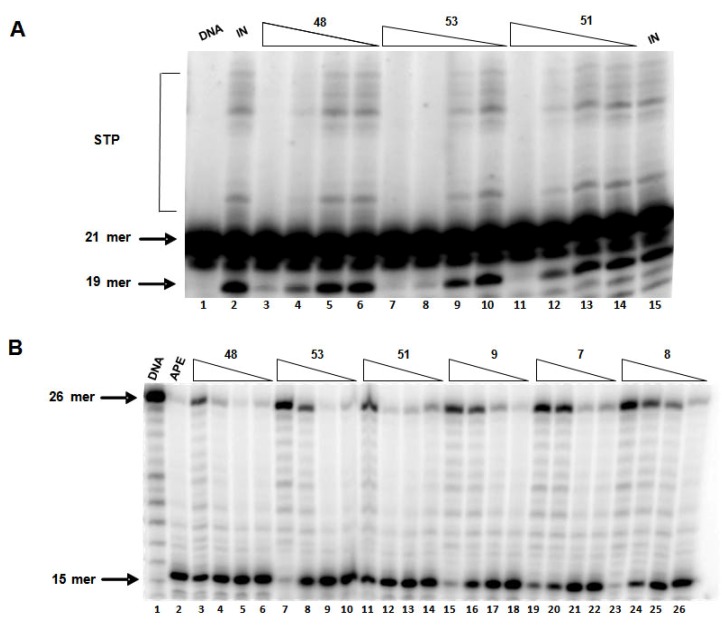
(A) A representative gel showing inhibition of purified IN by selected compounds. Lanes 1: DNA alone; Lanes 2 and 15: DNA and IN alone with no drug; Lanes 3–14: DNA with IN and selected drug concentrations (lanes: 3–6: compound **48**; lanes 7–10: compound **53**; lanes 11–14: compound **51**; at concentrations: 100, 33.3, 11.1 and 3.7 μM); (B) A representative gel showing inhibition of purified APE1 by selected compounds. Lanes 1: DNA alone; Lanes 2 and 15: DNA and APE1 alone with no drug; Lanes 3–26: DNA with APE1 and selected drug concentrations (lanes: 3–6: compound **48**; lanes 7–10: compound **53**; lanes 11–14: compound **51**; lanes 15–18: compound **9**; lanes 19–22: compound **7**; lanes 23–26: compound **8**; at concentrations: 100, 33.3, 11.1 and 3.7 μM).

In order to further understand the effect of electron-donating groups in ring A on activity, we synthesized analogs with hydroxyl groups at position 4. Here again, strong electron-withdrawing and hydrophobic substitutions in substructure B appears to influence IN inhibition (Table 5). The most potent IN inhibition was seen with compound **53** having 3,5-diiodophenol substitution (IC_50_ value of 7 ± 3 μM and 3 ± 2 for 3’-processing and strand transfer, respectively). Compared to compounds bearing trimethoxy substitution (Table 4), some of these compounds are more potent, suggesting that electron-donating substituents in ring A, together with strong electron-withdrawing and hydrophobic substitution in substructure B, leads to favorable IN inhibitory activity. Interestingly, substitution with a hydroxyl group in ring A improved selectivity towards strand transfer inhibition (Figure 3A). This may be due to possible formation of stabilizing hydrogen bonds at the IN active site. Overall, these rhodanine derivatives with an aliphatic thioxoamide linker have an improved IN inhibitory activity over those previously reported with an aliphatic linker [14].

**Table 5:** Inhibition of HIV-1 IN and APE1 catalytic activities by rhodanines **41**–**54**.

Compound	Structure	Inhibition of IN catalytic activity IC_50_ (µM)		Inhibition of APE1 catalytic activityIC_50_ (μM)
		3’-Processing	Strand Transfer	
**41**	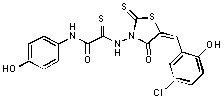	>100	67	>100
**42**	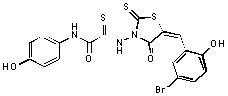	>100	>100	>100
**43**	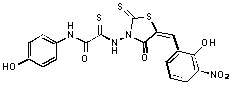	>100	88	>100
**44**	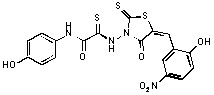	53 ± 8	45 ± 14	>100
**45**	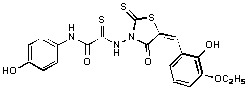	>100	>100	>100
**46**	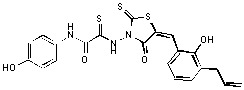	>100	100	>100
**47**	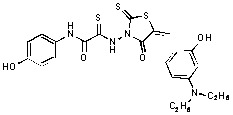	>100	>100	>100
**48**	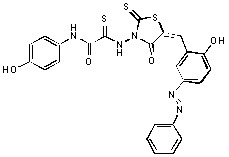	17 ± 7	8 ± 2	>100
**49**	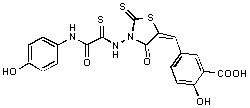	80	45	>100
**50**	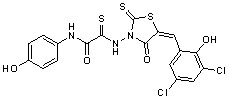	60	55	>100
**51**	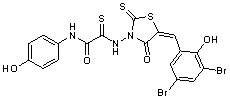	28 ± 1	21 ± 7	>100
**52**	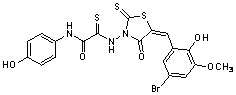	38 ± 27	23 ± 9	>100
**53**	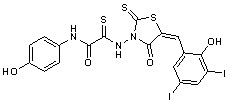	7 ± 3 (12.5)^a^	3 ± 2 (11)^a^	62 ± 3
**54**	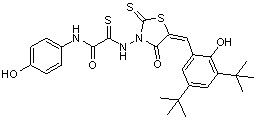	>100	>100	>100

^a^ Values in the parenthesis were obtained in the presence of Mg^2+^ as the cofactor.

### 2.3. In vitro APE1 Inhibitory Activity

The specificity of these rhodanine derivatives against IN was assessed by counter-screening against human apurinic/apyrimidinic endonuclease 1 (APE1), another DNA-binding enzyme. APE1 plays an important role in DNA repair by the base excision pathway. Like IN, APE1 possesses endonucleolytic activity and cleaves the phosphodiester DNA backbone 5’ to its recognition site. Like IN and other DNA-binding enzymes, optimal APE1 catalytic activity requires the presence of a divalent metal ion. APE1 is also an attractive target for designing cancer therapeutics [24,25,26]. 

Most compounds showed no inhibition against APE1 at the maximum tested concentration of 100 μM. Compound **53**, with the most potent IN inhibitory activity, exhibited only a weak APE1 inhibition with an IC_50_ value of 62 ± 3 μM. Its inhibitory activity is 20-fold more selective for IN than APE1. Similarly, compounds **48** and **51**, with promising IN inhibitory activity, did not show any significant APE1 inhibition at 100 μM. On the other hand, two compounds, **7** and **8**, showed a moderate to weak APE1 inhibition with IC_50_ values of 45 ± 51 μM and 76 ± 5 μM, respectively, while they lacked any IN inhibitory activity. Similarly, compound **9** inhibited APE1 activity with an IC_50_ value of 47 ± 2 μM (Figure 3B). However, it also exhibited an equal inhibitory effect on IN (IC_50_ value of 33 μM), suggesting possible similarities in its mechanism of action. 

### 2.4. Docking Studies

**Figure 4:**
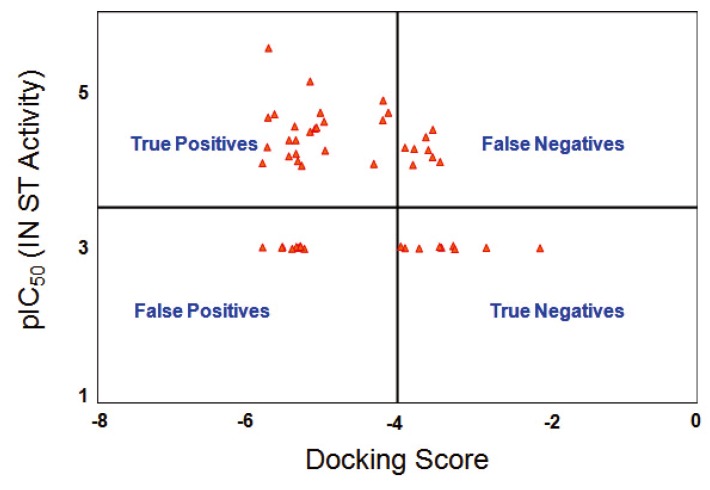
Plot of pIC_50_ (IN ST activity) versus the predicted Glide docking score.

To predict the possible binding modes and enzyme inhibition mechanism, compounds **6**–**54** were docked onto the active sites of both HIV-1 IN and APE1, using GOLD 4.0, the automated docking program, and Glide, the grid-based ligand docking with energetics software from Schrödinger. The docking scores for these compounds are reported in Table 6. A plot of the pIC_50_ (ST activity) versus the predicted Glide docking score of these 49 compounds against IN is shown in Figure 4. Although a significant correlation between docking scores and inhibitory activities of the compounds was not routinely observed, there was a general trend where many of the active compounds scored high. Of the 32 active compounds with a ST IC_50_ value less than 100 µM, 24 compounds were correctly predicted as true positives. Eight compounds, which were moderately active or least active, were predicted as false negatives. Similarly, out of the 18 inactive compounds with ST IC_50_ values greater than 100 µM, 9 compounds were correctly predicted as true negatives, while 9 inactive compounds were over predicted as false positives. We found no correlation between the APE1 inhibitory activity and the docking scores. 

**Table 6:** Comparison of docking scores of rhodanine-based compounds against HIV-1 IN and APE1.

Compound	HIV-1 IN ST IC_50_	APE1 IC_50_	Molecular Docking against HIV-IN		Molecular Docking against APE1	
	(μM)	(μM)	GOLD Fitness	Glide Score	GOLD Fitness	Glide Score
**6**	>100	>100	44.52	−3.26	54.65	−3.39
**7**	>100	45 ± 51	43.22	−3.42	56.72	−3.67
**8**	>100	76 ± 5	43.55	−3.24	50.35	−4.22
**9**	33	47 ± 2	42.9	−3.53	48.98	−3.60
**10**	>100	>100	44.44	−3.90	50.16	−5.63
**11**	20 ± 6	>100	41.15	−4.12	54.13	−5.31
**12**	93	89 ± 13	50.61	−3.80	55.33	−4.17
**13**	41 ± 27	93	56.35	−3.62	56.55	−4.44
**14**	58	>100	53.7	−3.78	61.16	−4.47
**15**	56 ± 11	−	50.97	−3.90	61.76	−3.90
**16**	74 ± 20	>100	49.48	−3.54	64.47	−3.21
**17**	60	>100	53.83	−3.59	51.32	−4.94
**18**	14 ± 5	>100	53.72	−4.19	48.2	−4.11
**19**	25 ± 11	>100	49.03	−4.20	48.36	−4.33
**20**	85 ± 21	>100	44.77	−3.44	57.11	−4.09
**21**	>100	>100	48.46	−3.71	53.61	−3.82
**22**	90	>100	36.49	−4.32	47.64	−4.67
**23**	>100	83	39.77	−3.44	52.31	−4.50
**24**	>100	>100	44.93	−3.96	50.75	−4.11
**25**	>100	>100	46.72	−2.82	49.01	−4.22
**26**	>100	−	47.85	−2.10	49.75	−4.46
**27**	20	>100	47.43	−5.03	46.7	−5.82
**28**	30 ± 19	>100	45.6	−5.37	47.93	−4.78
**29**	72 ± 23	>100	39.57	−5.45	49.09	−5.72
**30**	>100	>100	44.27	−5.33	49.84	−5.03
**31**	>100	>100	37.03	−5.24	47.14	−5.00
**32**	83 ± 3	>100	45.69	−5.33	47.69	−5.74
**33**	>100	>100	29.36	−5.29	39.69	−5.32
**34**	26 ± 14	>100	46.9	−4.98	52.47	−5.35
**35**	61 ± 11	>100	38.25	−4.97	47.61	−5.21
**36**	>100	>100	43.69	−5.35	46.27	−5.35
**37**	35 ± 6	>100	45.35	−5.17	49.67	−4.34
**38**	32 ± 11	65 ± 33	45.18	−5.10	49.09	−5.43
**39**	31 ± 16	>100	44.6	−5.08	50.75	−4.36
**40**	95	>100	21.11	−5.28	14.58	−3.79
**41**	67	>100	44.68	−5.35	52.5	−5.82
**42**	>100	>100	45.63	−5.29	49.24	−4.52
**43**	88	>100	50.44	−5.80	49.42	−6.18
**44**	45 ± 14	>100	44.75	−5.36	49.86	−5.08
**45**	>100	>100	42.79	−5.53	49.77	−5.20
**46**	100	>100	48.8	−5.80	52.75	−4.85
**47**	>100	>100	37.43	−5.54	45.29	−4.99
**48**	8 ± 2	>100	49.67	−5.16	59.85	−6.65
**49**	45	>100	44.68	−5.45	54.27	−7.48
**50**	55	>100	44.04	−5.74	51.34	−5.91
**51**	21 ± 7	>100	44.19	−5.64	54.26	−5.78
**52**	23 ± 9	>100	43.43	−5.73	50.25	−5.92
**53**	3 ± 2	62 ± 3	41.53	−5.72	55.04	−4.79
**54**	>100	>100	16.79	−5.40	17.8	−4.34

**Figure 5:**
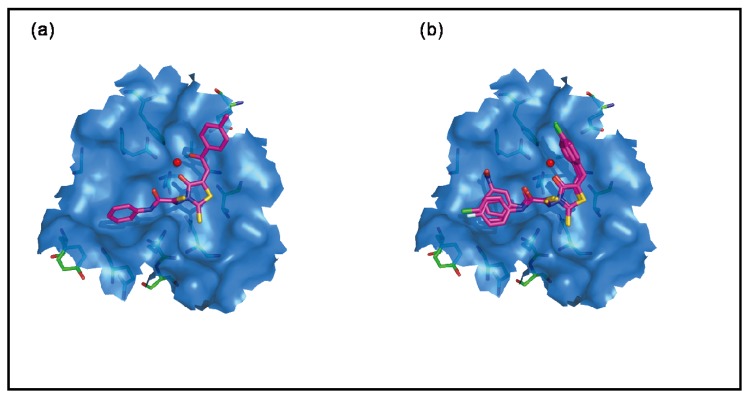
The proposed binding modes of **(a)** compound **11**, and **(b)** compound **6**–**8** in the HIV-1 IN active site. Close contact residues are shown as green sticks. Compounds **11** and **6**–**8** are shown in pink (colored by atom types) and Mg^2+^ is shown in red.

Compounds **6**–**8**, with a weak electron-withdrawing group on ring B, did not show any IN inhibitory activity at the highest tested concentration (Table 1). However, the introduction of a carbonyl group in substructure B (**11**) significantly improved strand transfer activity. Interestingly, docking simulations with Glide also estimated a high fitness score for this compound. The observed activity of the compound **11** supports the predicted binding interactions inside the IN active side. As shown in Figure 5, the oxygen atom of the rhodanine core and the oxygen atom of the substructure ring B are involved in interactions with both the Mg^2+^ ion and the active site residues D64 and D116. Additionally, the donor nitrogen atom of **11** forms a hydrogen bond with E152. The importance of this additional carbonyl of **11** in substructure B can further be understood by comparing against the activity and binding mode of compound **9** (Figure 5). A possible explanation for the lower activity of **9**, as compared to **11**, might be the lack of such interactions with key active residues D64 and D116 in the substructure B. Compounds **6**–**8** show a similar binding mode at the HIV-1 IN active site.

Compounds **12**–**21** with an electron-withdrawing nitrofuran ring in substructure B exhibit a moderate inhibition of strand IN transfer. Docking results predicted seven of these moderately active (**12**–**17** and **20**) compounds as false negatives, with their predicted activities lower than their actual activities. Compounds **27**–**40**, with electron-donating trimethoxy group on ring A, have shown moderate IN inhibitory activity. Docking results predicted four inactive compounds (**30**–**31**, **33** and **36**) as false positives, with their predicted activities being higher than their actual activities. 

**Figure 6:**
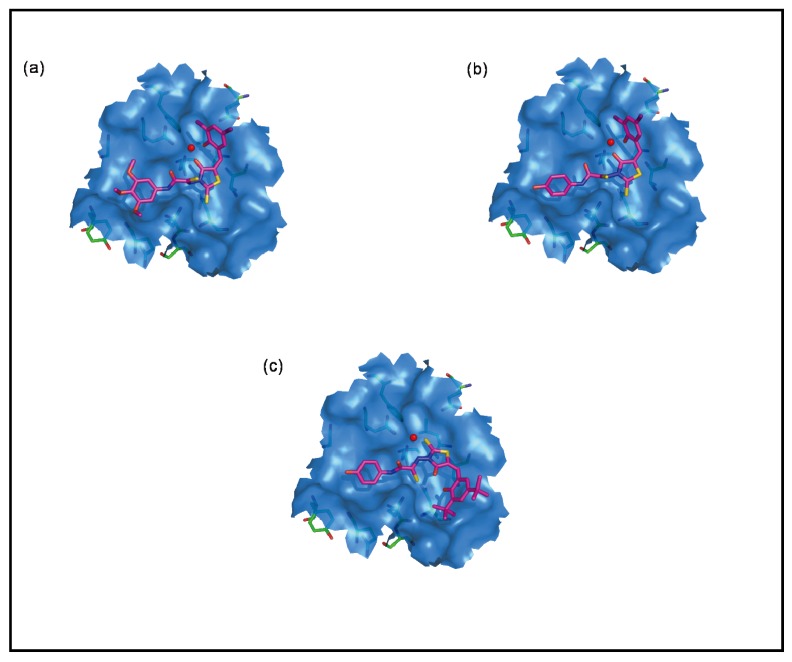
The proposed binding modes of (a) compound **39**; (b) compound **53**; and (c) compound **54** in the HIV-1 IN active site. Close contact residues are shown as green sticks. Compounds **39**, **53**, and **54** are shown in pink (colored by atom types) and Mg^2+^ is shown in red.

The binding mode of the most active compound **53** is shown in Figure 6. The residues in close contact with the compound **53** were D64, C65, T66, H67, D116, I141, Q148, E152, and N155. As shown in Figure 6, the carbonyl oxygen atom of the rhodanine and the hydroxyl oxygen atom in ring B are involved in interactions with both the Mg^2+^ ion and the amino acid residues D64 and C65. The two donor nitrogen atoms of **53** formed strong H-bonding interactions with the key active site residues D64 and E152. Additionally, the hydroxyl oxygen atom of the ring A was involved in a strong H-bonding interaction with the amino acid residue I141. The presence of these interactions with key active site residues explains the potency of **53**. A close analogue to **53** is compound **39**. The replacement of *p*-hydroxyl group (**53**) in ring A by a trimethoxy group (e.g., compound **39**) resulted in a 10-fold decrease in potency. The binding mode of compound **39** is shown in Figure 6. A possible explanation for its lower activity, as compared to **53**, might be the lack of H-bonding interactions with the amino acid residue I141. These docking results confirm the observations from the SAR studies. Indeed, the *p*-hydroxy group in ring A is able to form stabilizing H-bonds with I141. This accounts for the increased potency of compounds **41**–**54** as compared to their trimethoxy analogues **27**–**40**.

Orientation of the rhodanine core and substructure B are also important for IN inhibitory activity, contributing to the formation of key interactions with the metal ion and active site residues. Accordingly, replacement of the iodine groups on ring B by *t*-butyl groups (**54**) led to a drastic change in the binding pattern of ring B and a loss of activity. Though Glide estimated similar scores for both **53 **and **54**, differences in activity can be clearly explained from the GOLD docking poses (Figure 6). With the introduction of the bulky *t*-butyl groups, the rhodanine ring becomes inverted and no longer interacts with the Mg^2+^ ion and the active site residues. This might be responsible for the loss of activity of compound **54**.

### 2.5. Drug-like Properties

Oral bioavailability is a desirable property of compounds under investigation in the drug discovery process. Lipinski’s rule-of-five is a simple model to forecast the absorption and intestinal permeability of a compound [27,28]. In the rule-of-five model, compounds are considered likely to be well absorbed when they possess these attributes—molecular weight < 500, cLogP < 5, number of H-bond donors < 5, number H-bond acceptors < 10, and number of rotatable bonds < 10. Further, we calculated the polar surface area, which is also used to predict drug absorption [29,30]. Earlier studies by Palm *et al.* [31,32,33] and Kelder *et al.* [34] suggest that the compounds with a polar surface area >140 Å^2^ would tend to show poor (<10%) absorption, whereas the compounds with polar surface area <60 Å^2^ could be predicted to show complete (>90%) absorption. The calculated atom-based Log P (S + logP) values of most of the active compounds ranged from 2.0 to 5.0, and the H-bond donor and acceptor counts are ≤5 and <10, respectively. High lipophilicity is often an issue with many rhodanine-containing structures impeding their cell permeability and in vivo activity [22]. However, most of the compounds presented here have polar surface areas <140 Å^2^ and log P value of **<**5. Therefore, most of these analogs have favorable drug-like properties and are potentially interesting for further optimization.

**Table 7:** Predicted physicochemical and ADMET properties of rhodanine-based compounds.

Compound	MWt	MolVol	MlogP	S+logP	S+logD	S+Vd	S+BBB	PSA_2D	PolASA_3D	HBD	HBA
**6**	451.94	322	3.58	3.68	2.65	0.25	High	64	59	2	5
**7**	468.39	336	3.70	3.88	2.85	0.22	High	64	59	2	5
**8**	478.94	333	3.29	3.03	2.26	0.21	Low	106	130	2	7
**9**	463.97	347	2.92	3.53	2.46	0.27	High	73	55	2	6
**10**	438.54	322	2.25	2.93	2.13	0.26	Low	79	66	3	5
**11**	441.54	336	2.58	2.87	1.86	0.29	High	81	73	2	6
**12**	452.45	298	2.44	2.50	1.62	0.23	Low	119	127	2	8
**13**	468.90	312	2.56	2.67	1.93	0.23	Low	119	126	2	8
**14**	479.46	308	2.24	2.47	1.50	0.19	Low	162	236	2	10
**15**	502.46	326	2.91	2.90	1.95	0.25	Low	119	140	2	8
**16**	570.46	357	3.75	3.61	2.56	0.38	Low	119	140	2	8
**17**	464.49	322	1.83	2.37	1.43	0.18	Low	128	136	2	9
**18**	494.51	350	1.61	2.41	1.38	0.18	Low	137	137	2	10
**19**	524.54	378	1.12	2.33	1.25	0.18	Low	146	138	2	11
**20**	590.60	420	2.08	2.20	1.11	0.25	Low	176	174	2	13
**21**	516.52	343	2.54	1.97	1.02	0.21	Low	158	234	2	11
**22**	579.66	469	0.36	2.76	1.35	0.21	Low	117	58	2	11
**23**	645.72	511	1.34	2.82	1.19	0.19	Low	147	93	2	13
**24**	571.64	434	2.13	2.74	1.28	0.19	Low	129	126	2	11
**25**	557.58	417	2.45	3.73	2.24	0.29	Low	90	50	2	8
**26**	625.58	448	2.96	4.41	2.58	0.39	Low	90	48	2	8
**27**	540.02	410	1.39	3.41	1.86	0.21	Low	111	78	3	9
**28**	584.47	417	1.50	3.47	1.88	0.18	Low	111	94	3	9
**29**	550.58	406	1.10	2.99	1.14	0.19	Low	154	157	3	11
**30**	550.58	406	1.10	3.12	1.15	0.20	Low	154	161	3	11
**31**	549.63	441	0.87	2.98	1.53	0.19	Low	120	89	3	10
**32**	545.64	448	1.74	3.79	2.36	0.17	Low	111	93	3	9
**33**	576.70	490	1.29	3.41	2.31	0.23	Low	115	96	3	10
**34**	609.69	469	1.51	4.04	2.06	0.25	Low	134	90	3	11
**35**	549.59	413	0.27	3.10	0.60	0.20	Low	149	165	4	11
**36**	574.47	427	1.60	3.83	2.14	0.20	Low	111	73	3	9
**37**	663.37	441	1.81	3.88	1.96	0.19	Low	111	87	3	9
**38**	614.50	445	0.98	3.13	1.59	0.18	Low	120	92	3	10
**39**	757.37	455	2.02	4.17	2.16	0.20	Low	111	79	3	9
**40**	617.79	560	2.82	5.04	3.82	0.34	Low	111	73	3	9
**41**	465.94	333	2.21	2.97	1.70	0.23	Low	105	108	4	7
**42**	510.39	340	2.32	3.04	1.75	0.22	Low	105	107	4	7
**43**	476.50	329	1.85	2.84	1.22	0.18	Low	148	195	4	9
**44**	476.50	329	1.85	2.70	1.05	0.18	Low	148	206	4	9
**45**	475.55	364	1.68	2.89	1.69	0.21	Low	114	124	4	8
**46**	471.56	371	2.32	3.20	2.05	0.18	Low	105	122	4	7
**47**	502.62	413	2.13	3.23	2.33	0.26	Low	109	131	4	8
**48**	535.61	392	2.35	3.90	2.19	0.27	Low	128	121	4	9
**49**	475.51	336	1.05	2.62	0.49	0.22	Low	143	195	5	9
**50**	500.39	350	2.43	3.40	1.97	0.21	Low	105	102	4	7
**51**	589.29	364	2.66	3.46	1.89	0.20	Low	105	118	4	7
**52**	540.42	368	1.80	3.11	1.80	0.19	Low	114	127	4	8
**53**	683.29	378	2.87	3.67	1.98	0.22	Low	105	111	4	7
**54**	543.71	483	3.44	4.53	3.51	0.39	Low	105	104	4	7

MWt: Molecular weight; MolVol: Molal volume at normal boiling point; MlogP: Moriguchi octanol-water partition coefficient; S + logP: Simulation plus octanol-water partition coefficient; S + logD: Simulation plus octanol-water distribution coefficient; S + Vd: Simulation plus volume of distribution; S + BBB: Simulation plus BBB permeability; PSA_2D: 2D-polar surface area; PolASA_3D: 3D-polar solvent accessible surface area; HBD: Hydrogen bond donors; HBA: Hydrogen bond acceptors.

### 2.6. Cytotoxicity and Antiviral Activity

Several rhodanine structures such as the rhodacyanine dyes have been found to possess anticancer and antitumor properties. Hence, we evaluated the ability of these rhodanine-based compounds to inhibit the proliferation of various cancer cell lines. Compounds **13** and **17** were moderately active against a colon cancer cell line (HCT 116) and a pancreatic cell line (Panc-1) (GI_50_ values provided in Table 8). 

**Table 8:** *In vitro* cytotoxicity of rhodanine-based derivatives against a panel of cancer cell lines.

Compounds	GI_50_ (μM) ^a^				
	MDA-MD435	Panc-1	HT 29	HCT 116p53^+/+^	HCT 116p53^−/^^−^
**13**	13.5 ± 5.2	8.6 ± 1.6	>10	>10	6.5 ± 1.2
**17**	9 ± 1.1	8.2 ± 1.2	9.6	5.1 ± 0.8	6.1 ± 2.2

^a^ Values are reported as GI_50_ in μM, the concentration of the compound required to cause 50% inhibition of cell proliferation. GI_50_ values are calculated from at least three independent experiments at multiple doses.

The remaining compounds lacked any significant cytotoxicity at the maximum tested concentration of 10 μM. We also tested the antiviral activity of compounds **48**, **51** and **53** against HIV-1 in MT-4 cell infectivity assay. Unfortunately, these compounds did not show any significant antiviral activity. Optimization studies are underway to design rhodanines with more favorable physiochemical properties and improved antiviral activity.

## 3. Experimental

### 3.1. General

The ^1^H-NMR spectra were recorded on a Bruker AC spectrometer (200 MHz) using DMSO-d_6 _as solvent. The data are given in parts per million (ppm) and are referenced to an internal standard of tetramethylsilane (TMS, *δ* 0.00 ppm). The spin-spin coupling constants (*J*) are given in Hz. Peak multiplicity is reported as s (singlet), d (doublet), dd (double doublet), t (triplet), m (multiplet), and br s (broad singlet). The mass spectra were obtained on a Kratos instrument using a direct inlet system; the ionization energy was 70 eV; the accelerating voltage was 1.75 kV. Melting points were measured on a Boetius hot-stage apparatus. 5-Benzylidene-4-oxo-2-thioxothiazolidines **6**–**54** were puriﬁed by silica gel flash chromatography column (eluent: 3:1 petroleum ether-ethyl acetate). 2-Thioxo-1,3-thiazolidin-4-ones were prepared according to a previously reported procedure (Scheme 1) [23].

### 3.2. General Procedures for the Synthesis of 5-Benzylidene-4-oxo-2-thioxothiazolidine (6–54)

A flask containing methanol (10 mL) was charged with the appropriate rhodanine (0.13 mmol, 1.0 equivalents), the appropriate benzaldehyde (0.13 mmol, 1.0 equivalents), and ethylenediammonium diacetate (0.1 equivalents). The solution was stirred at room temperature for 1–2 h. The solvent was evaporated under reduced pressure and the product residue was purified by flash chromatography.

*2-{[5-(4-Chlorobenzylidene)-4-oxo-2-thioxo-1,3-thiazolidin-3-yl]amino}-N-(4-fluorophenyl)-2-thioxo-acetamide *(**6**). Yield 87%; m.p. 204–205 °C; ^1^H-NMR: *δ* 7.09 (d, 2H, H_arom_, *J* = 8.7 Hz), 7.55–7.7 (m, 7H, H_arom_, *J* = 8.3 Hz), 9.89 (s, 1H, NH); MS (EI) *m/z* (%): 541 [M^+^] (78). Anal. Calcd. for C_18_H_11_ClFN_3_O_2_S_3_: C, 47.84; H, 2.45; Cl, 7.84; F, 4.20; N, 9.30; O, 7.08; S, 21.28. Found: C, 47.80; H, 2.39; Cl, 7.88; N, 9.38; S, 21.20.

*2-{[5-(4-Chlorobenzylidene)-4-oxo-2-thioxo-1,3-thiazolidin-3-yl]amino}-N-(4-chlorophenyl)-2-thioxo-acetamide *(**7**). Yield 87%; m.p. 211–212 °C; ^1^H-NMR: *δ* 7.51−7.65 (m, 9H, H_arom_), 9.89 (s, 1H, NH); MS (EI) *m/z* (%) 468 [M^+^] (85). Anal. Calcd. for C_18_H_11_C_l2_N_3_O_2_S_3_: C, 46.16; H, 2.37; Cl, 15.14; N, 8.97; O, 6.83; S, 20.54. Found: C, 46.21; H, 2.30; Cl, 15.24; N, 8.91; S, 20.58.

*2-{[5-(4-Chlorobenzylidene)-4-oxo-2-thioxo-1,3-thiazolidin-3-yl]amino}-N-(3-nitrophenyl)-2-thioxo-acetamide *(**8**). Yield 71%; m.p. 221–222 °C; ^1^H-NMR: *δ* 7.55–7.65 (m, 5H, H_arom_), 7.77−7.81 (t, 1H, H_arom_), 7.94 (d, 1H, H_arom_, *J* = 8.2 Hz), 8.17 (d, 1H, H_arom_, *J* = 8 Hz), 8.62 (s, 1H, H_arom_), 9.89 (s, 1H, NH); MS (EI) *m/z* (%) 478 [M^+^] (62). Anal. Calcd. for C_18_H_11_ClN_4_O_4_S_3_: C, 45.14; H, 2.31; Cl, 7.40; N, 11.70; O, 13.36; S, 20.08. Found: C, 45.07; H, 2.28; Cl, 7.40; N, 11.79; S, 20.01.

*2-{[5-(4-Chlorobenzylidene)-4-oxo-2-thioxo-1,3-thiazolidin-3-yl]amino}-N-(4-methoxyphenyl)-2-thioxoacetamide* (**9**). Yield 90%; m.p. 186–187 °C; ^1^H-NMR: *δ* 3.77 (s, 3H, OCH_3_), 7.09 (d, 2H, H_arom_, *J* = 8.7 Hz), 7.51–7.66 (m, 6H, H_arom_), 9.89 (s, 1H, NH); MS (EI) *m/z* (%) 463 [M^+^] (93). Anal. Calcd. for C_19_H_14_ClN_3_O_3_S_3_: C, 49.18; H, 3.04; Cl, 7.64; N, 9.06; O, 10.34; S, 20.73. Found: C, 49.25; H, 3.14; Cl, 7.56; N, 9.26; S, 20.71.

*2-{[5-(1H-indol-3-ylmethylene)-4-oxo-2-thioxo-1,3-thiazolidin-3-yl]amino}-N-phenyl-2-thioxo-acetamide* (**10**). Yield 83%; m.p. 206–207 °C; ^1^H-NMR: *δ* 7.06–7.12 (m, 2H, H_arom_), 7.26–7.3 (m, 3H, H_arom_), 7.41 (d, 1H, H_arom_, *J* = 7.9 Hz), 7.63 (d, 2H, H_arom_, *J* = 7.3 Hz), 7.8 (s, 1H, CH), 8.07 (d, 1H, H_arom_, *J* = 7.8 Hz), 8.69 (s, 1H, H_arom_), 9.8 (s, 1H, NH); MS (EI) *m/z* (%) 438 [M^+^] (87). Anal. Calcd. for C_20_H_14_N_4_O_2_S_3_: C, 54.78; H, 3.22; N, 12.78; O, 7.30; S, 21.93. Found: C, 54.85; H, 3.29; N, 12.73; S, 21.85.

*2-{5-[2-(4-Methylphenyl)-2-oxoethylidene]-4-oxo-2-thioxo-1,3-thiazolidin-3-yl}amino-N-phenyl-2-thioxoacetamide* (**11**). Yield 65%; m.p. 147–148 °C; ^1^H-NMR: *δ* 2.43 (s, 3H, CH_3_), 7.08–7.13 (t, 1H, H_arom_, *J* = 7.3 Hz), 7.18-7.30 (m, 4H, H_arom_), 7.62 (d, 2H, H_arom_, *J* = 7.3 Hz), 7.68 (s, 1H, CH), 7.91 (d, 2H, H_arom_, *J* = 8.4 Hz), 9.89 (s, 1H, NH); MS (EI) *m/z* (%) 441 [M^+^] (82). Anal. Calcd. for C_19_H_13_N_3_O_3_S_3_: C, 53.38; H, 3.06; N, 9.83; O, 11.23; S, 22.50. Found: C, 53.46; H, 3.13; N, 9.76; S, 22.50.

*N-(4-fluorophenyl)-2-{5-[(5-nitro-2-furyl)methylene]-4-oxo-2-thioxo-1,3-thiazolidin-3-yl}amino-2-thioxoacetamide* (**12**). Yield 53%; m.p. 155–156 °C; ^1^H-NMR: *δ* 7.09 (d, 2H, H_arom_, *J* = 8.6 Hz), 7.59 (d, 1H, H_arom_, *J* = 3.6 Hz), 7.67–7.7 (m, 3H, H_arom_), 9.89 (s, 1H, NH); MS (EI) *m/z* (%) 452 [M^+^] (35). Anal. Calcd. for C_16_H_9_FN_4_O_5_S_3_: C, 42.47; H, 2.00; F, 4.20; N, 12.38; O, 17.68; S, 21.26. Found: C, 42.56; H, 1.89; N, 12.46; S, 21.18.

*N-(4-chlorophenyl)-2-{5-[(5-nitro-2-furyl)methylene]-4-oxo-2-thioxo-1,3-thiazolidin-3-yl}amino-2-thioxoacetamide* (**13**). Yield 58%; m.p. 159–160 °C; ^1^H-NMR: *δ* 7.11 (s, 1H, CH), 7.53–7.63 (m, 5H, H_arom_), 7.68 (d, 1H, H_arom_, *J* = 3.6 Hz), 9.89 (s, 1H, NH); MS (EI) *m/z* (%) 468 [M^+^] (30). Anal. Calcd. for C_16_H_9_ClN_4_O_5_S_3_: C, 40.98; H, 1.93; Cl, 7.56; N, 11.95; O, 17.06; S, 20.51. Found: C, 41.06; H, 1.90; Cl, 7.50; N, 12.04; S, 20.54.

*2-{-5-[(5-Nitro-2-furyl)methylene]-4-oxo-2-thioxo-1,3-thiazolidin-3-yl}amino-N-(3-nitrophenyl)-2-thioxoacetamide* (**14**). Yield 49%; m.p. 152–153 °C; ^1^H-NMR: *δ* 7.11 (s, 1H, CH), 7.59 (d, 1H, H_arom_, *J* = 3.6 Hz), 7.67 (d, 1H, H_arom_, *J* = 3.6 Hz), 7.77–7.81 (t, 1H, H_arom_, *J* = 7.9 Hz), 7.94 (d, 1H, H_arom_, *J* = 8.2 Hz), 8.17 (d, 1H, H_arom_, *J* = 8.0 Hz), 8.62 (s, 1H, H_arom_), 9.89 (s, 1H, NH); MS (EI) *m/z* (%) 479 [M^+^] (39). Anal. Calcd. for C_16_H_9_N_5_O_7_S_3_: C, 40.08; H, 1.89; N, 14.61; O, 23.36; S, 20.06. Found: C, 40.01; H, 1.83; N, 14.69; S, 20.09.

*2-{5-[(5-Nitro-2-furyl)methylene]-4-oxo-2-thioxo-1,3-thiazolidin-3-yl}amino-2-thioxo-N-[3-(trifluoro-methyl)phenyl]acetamide* (**15**). Yield 53%; m.p. 163–164 °C; ^1^H-NMR: *δ* 7.11 (s, 1H, CH), 7.44 (d, 1H, H_arom_, *J* = 7.8 Hz), 7.59 (d, 1H, H_arom_, *J* = 3.6 Hz), 7.64-7.68 (m, 2H, H_arom_), 7.87 (d, 1H, H_arom_, *J* = 7.7 Hz), 8.18 (s, 1H, H_arom_), 9.89 (s, 1H, NH); MS (EI) *m/z* (%) 502 [M^+^] (43). Anal. Calcd. for C_17_H_9_F_3_N_4_O_5_S_3_: C, 40.64; H, 1.81; F, 11.34; N, 11.15; O, 15.92; S, 19.14. Found: C, 40.69; H, 1.74; N, 11.21; S, 19.20.

*N-[3,5-bis(trifluoromethyl)phenyl]-2-{5-[(5-nitro-2-furyl)methylene]-4-oxo-2-thioxo-1,3-thiazolidin-3-yl}amino-2-thioxoacetamide *(**16**). Yield 55%; m.p. 195–196 °C; ^1^H-NMR: δ 7.11 (s, 1H, CH), 7.59 (d, 1H, H_arom_, J = 3.6 Hz), 7.67-7.72 (m, 2H, H_arom_) 8.31 (s, 2H, H_arom_), 9.89 (s, 1H, NH); MS (EI) m/z (%) 570 [M^+^] (25). Anal. Calcd. for C_18_H_8_F_6_N_4_O_5_S_3_: C, 37.90; H, 1.41; F, 19.98; N, 9.82; O, 14.02; S, 16.86. Found: C, 37.81; H, 1.37; N, 9.87; S, 16.81.

*N-(4-methoxyphenyl)-2-{5-[(5-nitro-2-furyl)methylene]-4-oxo-2-thioxo-1,3-thiazolidin-3-yl}amino-2-thioxoacetamide* (**17**). Yield 73%; m.p. 187–188 °C; ^1^H-NMR: *δ* 3.77 (s, 3H, OCH_3_), 7.09 (d, 2H, H_arom_, *J* = 8.8 Hz), 7.11 (s, 1H, CH), 7.51 (d, 2H, H_arom_, *J* = 8.8 Hz), 7.59 (d, 1H, H_arom_, *J* = 3.6 Hz), 7.68 (d, 1H, H_arom_, *J* = 3.6 Hz), 9.89 (s, 1H, NH); MS (EI) *m/z* (%) 464 [M^+^] (57). Anal. Calcd. for C_17_H_12_N_4_O_6_S_3_: C, 43.96; H, 2.60; N, 12.06; O, 20.67; S, 20.71. Found: C, 44.08; H, 2.67; N, 12.06; S, 20.63.

*N-(3,4-dimethoxyphenyl)-2-{5-[(5-nitro-2-furyl)methylene]-4-oxo-2-thioxo-1,3-thiazolidin-3-yl}amino-2-thioxoacetamide* (**18**). Yield 68%; m.p. 156–157 °C; ^1^H-NMR: *δ* 3.71 (s, 3H, OCH_3_), 3.81 (s, 3H, OCH_3_), 6.88 (s, 1H, H_arom_), 6.95 (d, 1H, H_arom_, *J* = 7.9 Hz), 7.11 (s, 1H, CH), 7.29 (d, 1H, H_arom_, *J* = 7.9 Hz), 7.59 (d, 1H, H_arom_, *J* = 3.6 Hz), 7.68 (d, 1H, H_arom_, *J* = 3.6 Hz), 9.89 (s, 1H, NH); MS (EI) *m/z* (%) 494 [M^+^] (67). Anal. Calcd. for C_18_H_14_N_4_O_7_S_3_: C, 43.72; H, 2.85; N, 11.33; O, 22.65; S, 19.45. Found: C, 43.81; H, 2.87; N, 11.30; S, 19.47.

*2-{5-[(5-Nitro-2-furyl)methylene]-4-oxo-2-thioxo-1,3-thiazolidin-3-yl}amino-2-thioxo-N-(3,4,5-tri-methoxyphenyl)acetamide* (**19**). Yield 68%; m.p. 201–202 °C; ^1^H-NMR: *δ* 3.8 (s, 6H, OCH_3_), 3.86 (s, 3H, OCH_3_), 6.58 (s, 2H, H_arom_), 7.11 (s, 1H, CH), 7.59 (d, 1H, H_arom_, *J* = 3.6 Hz), 7.68 (d, 1H, H_arom_, *J* = 3.6 Hz), 9.9 (s, 1H, NH); MS (EI) *m/z* (%) 524 [M^+^] (75). Anal. Calcd. for C_19_H_16_N_4_O_8_S_3_: C, 43.51; H, 3.07; N, 10.68; O, 24.40; S, 18.34. Found: C, 43.57; H, 3.12; N, 10.65; S, 18.30.

*N-[2,5-diethoxy-4-(1H-tetrazol-1-yl)phenyl]-2-{5-[(5-nitro-2-furyl)methylene]-4-oxo-2-thioxo-1,3-thiazolidin-3-yl}amino-2-thioxoacetamide* (**20**). Yield 43%; m.p. 136–138 °C; ^1^H-NMR: δ 1.4–1.47 (t, 6H, CH_3_, J = 6.9 Hz), 4.13–4.21 (q, 4H, CH_2_, J = 6.7 Hz), 7.11 (s, 1H, CH), 7.24 (s, 1H, H_arom_), 7.32 (s, 1H, H_arom_),7.59 (d, 1H, H_arom_, J = 3.6 Hz), 7.68 (d, 1H, H_arom_, J = 3.6 Hz), 8.96 (s, 1H, H_arom_), 9.89 (s, 1H, NH); MS (EI) m/z (%) 590 [M^+^] (15). Anal. Calcd. for C_21_H_18_N_8_O_7_S_3_: C, 42.71; H, 3.07; N, 18.97; O, 18.96; S, 16.29. Found: C, 42.65; H, 3.01; N, 19.11; S, 16.22.

*N-[3-methyl-4-(1H-tetrazol-1-yl)phenyl]-2-{5-[(5-nitro-2-furyl)methylene]-4-oxo-2-thioxo-1,3-thia-zolidin-3-yl}amino-2-thioxoacetamide *(**21**). Yield 40%; m.p. 161–162 °C; ^1^H-NMR: δ 2.48 (s, 3H, CH_3_), 7.11 (s, 1H, CH), 7.59 (d, 1H, H_arom_, J = 3.6 Hz), 7.63-7.68 (m, 2H, H_arom_), 7.83 (d, 1H, H_arom_, J = 8.6 Hz), 8.01 (s, 1H, H_arom_), 8.95 (s, 1H, H_arom_), 9.89 (s, 1H, NH); MS (EI) m/z (%) 516 [M^+^] (33). Anal. Calcd. for C_18_H_12_N_8_O_5_S_3_: C, 41.86; H, 2.34; N, 21.69; O, 15.49; S, 18.62. Found: C, 41.91; H, 2.29; N, 21.77; S, 18.58.

*2-{[4-Oxo-2-thioxo-5-(3,4,5-trimethoxybenzylidene)-1,3-thiazolidin-3-yl]amino}-2-thioxo-N-(3,4,5-tri-methoxyphenyl)acetamide* (**22**). Yield 83%; m.p. 196–197 °C; ^1^H-NMR: *δ* 3.73 (s, 6H, CH_3_), 3.80 (s, 6H, CH_3_), 3.83 (s, 3H, CH_3_), 3.85 (s, 3H, CH_3_), 6.58 (s, 2H, H_arom_), 7.11 (s, 2H, H_arom_), 7.42 (s, 1H, CH), 9.89 (s, 1H, NH); MS (EI) *m/z* (%) 579 [M^+^] (76). Anal. Calcd. for C_24_H_25_N_3_O_8_S_3_: C, 49.73; H, 4.35; N, 7.25; O, 22.08; S, 16.59. Found: C, 49.81; H, 4.42; N, 7.19; S, 16.64.

*N-[2,5-diethoxy-4-(1H-tetrazol-1-yl)phenyl]-2-{[4-oxo-2-thioxo-5-(3,4,5-trimethoxybenzylidene)-1,3-thiazolidin-3-yl]amino}-2-thioxoacetamide* (**23**). Yield 83%; m.p. 158–159 °C; ^1^H-NMR: *δ* 1.4–1.47 (t, 3H, CH_3_, *J* = 6.9 Hz), 3.73 (s, 6H, CH_3_), 3.84 (s, 3H, CH_3_), 4.12–4.21 (q, 2H, CH_2_, *J* = 6.7 Hz), 7.11 (s, 2H, H_arom_), 7.24 (s, 1H, H_arom_), 7.32 (s, 1H, H_arom_), 7.42 (s, 1H, CH), 8.96 (s, 1H, H_arom_), 9.89 (s, 1H, NH); MS (EI) *m/z* (%) 645 [M^+^] (12). Anal. Calcd. for C_26_H_27_N_7_O_7_S_3_: C, 48.36; H, 4.21; N, 15.18; O, 17.34; S, 14.90. Found: C, 48.38; H, 4.15; N, 15.28; S, 14.85.

*N-[3-methyl-4-(1H-tetrazol-1-yl)phenyl]-2-{[4-oxo-2-thioxo-5-(3,4,5-trimethoxybenzylidene)-1,3-thiazolidin-3-yl]amino}-2-thioxoacetamide* (**24**). Yield 77%; m.p. 173–174 °C; ^1^H-NMR: *δ* 2.48 (s, 3H, CH_3_), 3.73 (s, 6H, CH_3_), 3.84 (s, 3H, CH_3_), 7.11 (s, 2H, H_arom_), 7.42 (s, 1H, CH), 7.64 (d, 1H, H_arom_, *J* = 8.6 Hz), 7.82 (d, 1H, H_arom_, *J* = 8.6 Hz), 8.01 (s, 1H, H_arom_), 8.95 (s, 1H, H_arom_), 9.89 (s, 1H, NH); MS (EI) *m/z* (%) 571 [M^+^] (23). Anal. Calcd. for C_23_H_21_N_7_O_5_S_3_: C, 48.33; H, 3.70; N, 17.15; O, 13.99; S, 16.83. Found: C, 48.39; H, 3.74; N, 17.24; S, 16.78.

*2-{[4-Oxo-2-thioxo-5-(3,4,5-trimethoxybenzylidene)-1,3-thiazolidin-3-yl]amino}-2-thioxo-N-[3-(tri-fluoromethyl)phenyl]acetamide* (**25**). Yield 89%; m.p. 160–161 °C; ^1^H-NMR: δ 3.73 (s, 6H, CH_3_), 3.84 (s, 3H, CH_3_), 7.11 (s, 2H, H_arom_), 7.42-7.44 (m, 2H), 7.64-7.68 (t, 1H, H_arom_, J = 7.8 Hz), 7.88 (d, 1H, H_arom_, J = 7.7 Hz), 8.17 (s, 1H, H_arom_), 9.89 (s, 1H, NH); MS (EI) m/z (%) 557 [M^+^] (58). Anal. Calcd. for C_22_H_18_F_3_N_3_O_5_S_3_: C, 47.39; H, 3.25; F, 10.22; N, 7.54; O, 14.35; S, 17.25. Found: C, 47.43; H, 3.19; N, 7.50; S, 17.27.

*N-[3,5-bis(trifluoromethyl)phenyl]-2-{[4-oxo-2-thioxo-5-(3,4,5-trimethoxybenzylidene)-1,3-thiazol-idin-3-yl]amino}-2-thioxoacetamide* (**26**). Yield 72%; m.p. 187–188 °C; ^1^H-NMR: δ 3.73 (s, 6H, CH_3_), 3.84 (s, 3H, CH_3_), 7.11 (s, 2H, H_arom_), 7.42 (s, 1H, CH), 7.7 (s, 1H, H_arom_), 8.3 (s, 2H, H_arom_), 9.89 (s, 1H, NH); MS (EI) m/z (%) 625 [M^+^] (17). Anal. Calcd. for C_23_H_17_F_6_N_3_O_5_S_3_: C, 44.16; H, 2.74; F, 18.22; N, 6.72; O, 12.79; S, 15.38. Found: C, 44.09; H, 2.78; N, 6.67; S, 15.35.

*2-{[5-(5-Chloro-2-hydroxybenzylidene)-4-oxo-2-thioxo-1,3-thiazolidin-3-yl]amino}-2-thioxo-N-(3,4,5-trimethoxyphenyl)acetamide* (**27**). Yield 83%; m.p. 216–217 °C; ^1^H-NMR: *δ* 3.80 (s, 6H, CH_3_), 3.86 (s, 3H, CH_3_), 6.58 (s, 2H, H_arom_), 7.04 (d, 1H, H_arom_, *J* = 8.9 Hz), 7.21 (d, 1H, H_arom_, *J* = 9.0 Hz), 7.73 (s, 1H, H_arom_), 7.81 (s, 1H, CH), 9.34 (s, 1H, OH), 10.71 (s, 1H, NH); MS (EI) *m/z* (%) 540 [M^+^] (89). Anal. Calcd. for C_21_H_18_ClN_3_O_6_S_3_: C, 46.71; H, 3.36; Cl, 6.56; N, 7.78; O, 17.78; S, 17.81. Found: C, 46.80; H, 3.41; Cl, 6.60; N, 7.69; S, 17.77.

*2-{[5-(5-Bromo-2-hydroxybenzylidene)-4-oxo-2-thioxo-1,3-thiazolidin-3-yl]amino}-2-thioxo-N-(3,4,5-trimethoxyphenyl)acetamide* (**28**). Yield 87%; m.p. 208–209 °C; ^1^H-NMR: *δ* 3.80 (s, 6H, CH_3_), 3.86 (s, 3H, CH_3_), 6.58 (s, 2H, H_arom_), 6.78 (d, 1H, H_arom_, *J* = 8.5 Hz), 7.05 (d, 1H, H_arom_, *J* = 8.3 Hz), 7.77 (s, 1H, H_arom_), 7.85 (s, 1H, CH), 9.16 (s, 1H, OH), 10.38 (s, 1H, NH); MS (EI) *m/z* (%) 584 [M^+^] (89). Anal. Calcd. for C_21_H_18_BrN_3_O_6_S_3_: C, 43.15; H, 3.10; Br, 13.67; N, 7.19; O, 16.42; S, 16.4. Found: C, 43.19; H, 3.13; Br, 13.62; N, 7.11; S, 16.44.

*2-{[5-(2-Hydroxy-3-nitrobenzylidene)-4-oxo-2-thioxo-1,3-thiazolidin-3-yl]amino}-2-thioxo-N-(3,4,5-trimethoxyphenyl)acetamide* (**29**). Yield 62%; m.p. 193–194 °C; ^1^H-NMR: *δ* 3.80 (s, 6H, CH_3_), 3.86 (s, 3H, CH_3_), 6.58 (s, 2H, H_arom_), 7.21–7.25 (t, 1H, H_arom_, *J* = 7.9 Hz), 7.87 (d, 1H, H_arom_, *J* = 7.7 Hz), 8.01 (s, 1H, CH), 10.26 (s, 1H, OH), 11.83 (s, 1H, NH); MS (EI) *m/z* (%) 550 [M^+^] (43). Anal. Calcd. for C_21_H_18_N_4_O_8_S_3_: C, 45.81; H, 3.30; N, 10.18; O, 23.25; S, 17.47. Found: C, 45.86; H, 3.32; N, 10.25; S, 17.50.

*2-{[5-(2-Hydroxy-5-nitrobenzylidene)-4-oxo-2-thioxo-1,3-thiazolidin-3-yl]amino}-2-thioxo-N-(3,4,5-trimethoxyphenyl)acetamide* (**30**). Yield 52%; m.p. 205–206 °C; ^1^H-NMR: *δ* 3.80 (s, 6H, CH_3_), 3.86 (s, 3H, CH_3_), 6.58 (s, 2H, H_arom_), 7.23 (d, 1H, H_arom_, *J* = 9.3 Hz), 7.91 (d, 1H, H_arom_, *J* = 9.0 Hz), 8.02 (s, 1H, CH), 8.19 (s, 1H, H_arom_), 8.96 (s, 1H, CH), 9.16 (s, 1H, OH), 10.81 (s, 1H, NH); MS (EI) *m/z* (%) 550 [M^+^] (43). Anal. Calcd. for C_21_H_18_N_4_O_8_S_3_: C, 45.81; H, 3.30; N, 10.18; O, 23.25; S, 17.47. Found: C, 45.78; H, 3.35; N, 10.23; S, 17.42.

*2-{[5-(3-Ethoxy-2-hydroxybenzylidene)-4-oxo-2-thioxo-1,3-thiazolidin-3-yl]amino}-2-thioxo-N-(3,4,5-trimethoxyphenyl)acetamide* (**31**). Yield 90%; m.p. 231–232 °C; ^1^H-NMR: *δ* 1.40–1.43 (t, 3H, CH_3_, *J* = 7.15 Hz), 3.80 (s, 6H, CH_3_), 3.86 (s, 3H, CH_3_), 4.04–4.09 (q, 2H, CH_2_), 6.58 (s, 2H, H_arom_), 6.76 (d,1H, H_arom_, *J* = 7.8 Hz), 7.17–7.21 (t, 1H, H_arom_, *J* = 7.8 Hz), 7.41 (d, 1H, H_arom_, *J* = 7.8 Hz), 8.25 (s, 1H, CH), 9.88 (s, 1H, OH), 11.45 (s, 1H, NH); MS (EI) *m/z* (%) 550 [M^+^] (43). Anal. Calcd. for C_23_H_23_N_3_O_7_S_3_: C, 50.26; H, 4.22; N, 7.64; O, 20.38; S, 17.50. Found: C, 50.36; H, 4.29; N, 7.58; S, 17.52.

*2-{[5-(3-Allyl-2-hydroxybenzylidene)-4-oxo-2-thioxo-1,3-thiazolidin-3-yl]amino}-2-thioxo-N-(3,4,5-trimethoxyphenyl)acetamide* (**32**). Yield 75%; m.p. 174–175 °C; ^1^H-NMR: *δ* 3.35–3.38 (m, 2H, H_alk_), 3.80 (s, 6H, OCH_3_), 3.86 (s, 3H, OCH_3_), 5.09–5.12 (m, 1H, H_alk_), 5.20–5.25 (m, 1H, H_alk_), 6.12–6.22 (m, 1H, H_alk_), 6.50 (s, 2H, H_arom_), 7.0 (d, 1H, H_arom_, *J *= 7.7 Hz), 7.11–7.15 (t, 1H, H_arom_, *J* = 7.7 Hz), 7.52 (d, 1H, H_arom_, *J* = 7.7 Hz), 8.25 (s, 1H, CH), 8.81 (s, 1H, OH), 10.21 (s, 1H, NH); MS (EI) *m/z* (%) 545 [M^+^] (32). Anal. Calcd. for C_24_H_23_N_3_O_6_S_3_: C, 52.83; H, 4.25; N, 7.70; O, 17.59; S, 17.63. Found: C, 52.91; H, 4.29; N, 7.66; S, 17.60.

*2-({5-[4-(Diethylamino)-2-hydroxybenzylidene]-4-oxo-2-thioxo-1,3-thiazolidin-3-yl}amino)-2-thioxo-N-(3,4,5-trimethoxyphenyl)acetamide* (**33**). Yield 87%; m.p. 210–211 °C; ^1^H-NMR: δ 1.07–1.12 (t, 6H, CH_3_, J = 7.2 Hz), 3.23–3.30 (q, 4H, CH_2_), 3.80 (s, 6H, OCH_3_), 3.86 (s, 3H, OCH_3_), 6.50 (s, 1H, H_arom_), 6.58–6.61 (m, 3H, H_arom_), 7.31 (d, 1H, H_arom_, J = 9.0 Hz), 8.28 (s, 1H, CH), 9.18 (s, 1H, OH), 10.85 (s, 1H, NH); MS (EI) m/z (%) 576 [M^+^] (72). Anal. Calcd. for C_25_H_28_N_4_O_6_S_3_: C, 52.07; H, 4.89; N, 9.71; O, 16.65; S, 16.68. Found: C, 52.17; H, 4.93; N, 9.76; S, 16.64.

*2-[(5-{2-Hydroxy-5-[phenyldiazenyl]benzylidene}-4-oxo-2-thioxo-1,3-thiazolidin-3-yl)amino]-2-thioxo-N-(3,4,5-trimethoxyphenyl)acetamide *(**34**). Yield 52%; m.p. 149–150 °C; ^1^H-NMR: δ 3.80 (s, 6H, OCH_3_), 3.86 (s, 3H, OCH_3_), 6.58 (s, 2H, H_arom_), 6.73 (d, 2H, H_arom_, J = 7.8 Hz), 7.0−7.04 (t, 1H, H_arom_, J = 7.3 Hz), 7.11-7.16 (m, 3H, H_arom_), 7.56 (s, 1H, H_arom_), 7.68 (d, 1H, H_arom_, J = 8.7 Hz), 8.31 (s, 1H, CH), 8.98 (s, 1H, OH), 10.05 (s, 1H, NH); MS (EI) m/z (%) 609 [M^+^] (21). Anal. Calcd. for C_27_H_23_N_5_O_6_S_3_: C, 53.19; H, 3.80; N, 11.49; O, 15.74; S, 15.78. Found: C, 53.27; H, 3.84; N, 11.49; S, 15.75.

*2-Hydroxy-5-{([4-oxo-3-({2-oxo-2-[(3,4,5-trimethoxyphenyl)amino]-ethanethioyl}amino)-2-thioxo-1,3-thiazolidin-5-ylidene]methyl}benzoic acid* (**35**). Yield 81%; m.p. 239–240 °C; ^1^H-NMR: δ 3.80 (s, 6H, OCH_3_), 3.86 (s, 3H, OCH_3_), 6.59 (s, 2H, H_arom_), 7.19 (d, 1H, H_arom_, J = 8.6 Hz), 7.82 (d, 1H, H_arom_, J = 8.5 Hz), 8.01 (s, 1H, CH), 8.17 (s, 1H, H_arom_), 10.35 (s, 2H, OH), 11.53 (s, 1H, NH); MS (EI) m/z (%) 549 [M^+^] (77). Anal. Calcd. for C_22_H_19_N_3_O_8_S_3_: C, 48.08; H, 3.48; N, 7.65; O, 23.29; S, 17.50. Found: C, 48.13; H, 3.53; N, 7.61; S, 17.48.

*2-{[5-(3,5-Dichloro-2-hydroxybenzylidene)-4-oxo-2-thioxo-1,3-thiazolidin-3-yl]amino}-2-thioxo-N-(3,4,5-trimethoxyphenyl)acetamide* (**36**). Yield 59%; m.p. 215–216 °C; ^1^H-NMR: δ 3.80 (s, 6H, OCH_3_), 3.86 (s, 3H, OCH_3_), 6.58 (s, 2H, H_arom_), 7.37 (s, 1H, H_arom_), 7.92 (s, 1H, H_arom_), 8.09 (s, 1H, CH), 10.89 (s, 1H, OH), 11.71 (s, 1H, NH); MS (EI) m/z (%) 574 [M^+^] (89). Anal. Calcd. for C_21_H_17_Cl_2_N_3_O_6_S_3_: C, 43.91; H, 2.98; Cl, 12.34; N, 7.31; O, 16.71; S, 16.74. Found: C, 43.90; H, 2.95; Cl, 12.41; N, 7.26; S, 16.72.

*2-{[5-(3,5-Dibromo-2-hydroxybenzylidene)-4-oxo-2-thioxo-1,3-thiazolidin-3-yl]amino}-2-thioxo-N-(3,4,5-trimethoxyphenyl)acetamide* (**37**). Yield 62%; m.p. 221–222 °C; ^1^H-NMR: δ 3.80 (s, 6H, OCH_3_), 3.86 (s, 3H, OCH_3_), 6.58 (s, 2H, H_arom_), 7.58 (s, 1H, H_arom_), 7.73 (s, 1H, H_arom_), 8.17 (s, 1H, CH), 10.26 (s, 1H, OH), 11.11 (s, 1H, NH); MS (EI) m/z (%) 663 [M^+^] (71). Anal. Calcd. for C_21_H_17_Br_2_N_3_O_6_S_3_: C, 38.02; H, 2.58; Br, 24.09; N, 6.33; S, 14.50.

*2-{[5-(5-Bromo-2-hydroxy-3-methoxybenzylidene)-4-oxo-2-thioxo-1,3-thiazolidin-3-yl]amino}-N-(3,4,5-trimethoxyphenyl)acetamide* (**38**). Yield 69%; m.p. 203–204 °C; ^1^H-NMR: *δ* 3.76 (s, 3H, OCH_3_), 3.80 (s, 6H, OCH_3_), 3.86 (s, 3H, OCH_3_), 6.58 (s, 2H, H_arom_), 6.88 (s, 1H, H_arom_), 7.43 (s, 1H, H_arom_), 8.20 (s, 1H, CH), 9.88 (s, 1H, OH), 10.95 (s, 1H, NH); MS (EI) *m/z* (%) 614 [M^+^] (65). Anal. Calcd. for C_22_H_20_BrN_3_O_7_S_3_: C, 43.00; H, 3.28; Br, 13.00; N, 6.84; S, 15.65.

*2-{[5-(2-Hydroxy-3,5-diiodobenzylidene)-4-oxo-2-thioxo-1,3-thiazolidin-3-yl]amino}-N-(3,4,5-trimethoxyphenyl)-2-thioxoacetamide *(**39**). Yield 60%; m.p. 235–236 °C; ^1^H-NMR: δ 3.80 (s, 6H, OCH_3_), 3.86 (s, 3H, OCH_3_), 6.58 (s, 2H, H_arom_), 7.42 (s, 1H, H_arom_), 7.83 (s, 1H, H_arom_), 8.09 (s, 1H, CH), 10.26 (s, 1H, OH), 11.33 (s, 1H, NH); MS (EI) m/z (%) 757 [M^+^] (21). Anal. Calcd. for C_21_H_17_I_2_N_3_O_6_S_3_: C, 33.30; H, 2.26; I, 33.51; N, 5.55; S, 12.68.

*2-{[5-(2-Hydroxy-3,5-diisopropylbenzylidene)-4-oxo-2-thioxo-1,3-thiazolidin-3-yl]amino}-N-(3,4,5-trimethoxyphenyl)-2-thioxoacetamide *(**40**). Yield 71%; m.p. 189–190 °C; ^1^H-NMR: δ 1.1–1.15 m, 12H, H_alk_), 2.43-2.54 (m, 1H, H_alk_), 3.14–3.22 (m, 1H, H_alk_), 3.80 (s, 6H, OCH_3_), 3.86 (s, 3H, OCH_3_), 6.58 (s, 2H, H_arom_), 6.8 (s, 1H, H_arom_), 7.16 (s, 1H, H_arom_), 7.67 (s, 1H, CH), 8.82 (s, 1H, OH), 10.11 (s, 1H, NH); MS (EI) m/z (%) 589 [M^+^] (45). Anal. Calcd. For C_27_H_31_N_3_O_6_S_3_: C, 54.99; H, 5.30; N, 7.12; S, 16.31.

*2-{[5-(5-Chloro-2-hydroxybenzylidene)-4-oxo-2-thioxo-1,3-thiazolidin-3-yl]amino}-N-(4-hydroxy-phenyl)-2-thioxoacetamide* (**41**). Yield 86%; m.p. 220–221 °C; ^1^H-NMR: *δ* 6.89 (d, 2H, H_arom_, *J* = 9 Hz), 7.04 (d, 1H, H_arom_, *J* = 8.9 Hz), 7.21 (d, 1H, H_arom_, *J* = 8.9 Hz), 7.41 (d, 2H, H_arom_, *J* = 9 Hz), 7.73 (s, 1H, CH), 7.79 (s, 1H, H_arom_), 8.25 (s, 2H, OH), 10.31 (s, 1H, NH); MS (EI) *m/z* (%) 465 [M^+^] (99). Anal. Calcd. for C_18_H_12_ClN_3_O_4_S_3_: C, 46.40; H, 2.60; Cl, 7.61; N, 9.02; O, 13.73; S, 20.64. Found: C, 46.37; H, 2.64; Cl, 7.60; N, 8.97; S, 20.64.

*2-{[5-(5-Bromo-2-hydroxybenzylidene)-4-oxo-2-thioxo-1,3-thiazolidin-3-yl]amino}-N-(4-hydroxy-phenyl)-2-thioxoacetamide* (**42**). Yield 79%; m.p. 190–191 °C; ^1^H-NMR: *δ* 6.78 (d, 1H, H_arom_, *J* = 8.5 Hz), 6.89 (d, 2H, H_arom_, *J* = 9 Hz), 7.06 (d, 1H, H_arom_, *J* = 8.5 Hz), 7.43 (d, 2H, H_arom_, *J* = 9 Hz), 7.77 (s, 1H, CH), 7.82 (s, 1H, H_arom_), 8.11 (s, 2H, OH), 10.17 (s, 1H, NH); MS (EI) *m/z* (%) 510 [M^+^] (84). Anal. Calcd. for C_18_H_12_BrN_3_O_4_S_3_: C, 42.36; H, 2.37; Br, 15.65; N, 8.23; O, 12.54; S, 18.85. Found: C, 42.36; H, 2.45; Br, 15.62; N, 8.20; S, 18.81.

*2-{[5-(2-Hydroxy-3-nitrobenzylidene)-4-oxo-2-thioxo-1,3-thiazolidin-3-yl]amino}-N-(4-hydroxy-phenyl)-2-thioxoacetamide* (**43**). Yield 66%; m.p. 179–180 °C; ^1^H-NMR: *δ* 6.88 (d, 2H, H_arom_, *J* = 9 Hz), 7.21–7.25 (t, 1H, H_arom_, *J* = 7.7 Hz), 7.42 (d, 2H, H_arom_, *J* = 9 Hz), 7.87 (d, 2H, H_arom_, *J* = 7.7 Hz), 8.01 (s, 1H, CH), 8.94 (s, 2H, OH), 10.76 (s, 1H, NH); MS (EI) *m/z* (%) 476 [M^+^] (84). Anal. Calcd. for C_18_H_12_N_4_O_6_S_3_: C, 45.37; H, 2.54; N, 11.76; O, 20.15; S, 20.19. Found: C, 45.35; H, 2.60; N, 11.82; S, 20.17.

*2-{[5-(2-Hydroxy-5-nitrobenzylidene)-4-oxo-2-thioxo-1,3-thiazolidin-3-yl]amino}-N-(4-hydroxy-phenyl)-2-thioxoacetamide* (**44**). Yield 69%; m.p. 199–200 °C; ^1^H-NMR: *δ* 6.89 (d, 2H, H_arom_, *J* = 9 Hz), 7.23 (d, 1H, H_arom_, *J* = 9.3 Hz), 7.43 (d, 2H, H_arom_, *J* = 8.8 Hz), 7.91 (d, 1H, H_arom_, *J* = 9.3 Hz), 8.01 (s, 1H, CH), 8.11 (s, 2H, OH), 8.2 (s, 1H, H_arom_), 10.05 (s, 1H, NH); MS (EI) *m/z* (%) 476 [M^+^] (73). Anal. Calcd. for C_18_H_12_N_4_O_6_S_3_: C, 45.37; H, 2.54; N, 11.76; O, 20.15; S, 20.19. Found: C, 45.33; H, 2.60; N, 11.81; S, 20.16.

*2-{[5-(3-Ethoxy-2-hydroxybenzylidene)-4-oxo-2-thioxo-1,3-thiazolidin-3-yl]amino}-N-(4-hydroxyphenyl)-2-thioxoacetamide* (**45**). Yield 90%; m.p. 184–185 °C; ^1^H-NMR: *δ* 1.4–1.43 (t, 3H, CH_3_, *J* = 6.9 Hz), 4.07 (q, 2H, CH_2_, *J* = 6.9 Hz), 6.75 (d, 1H, H_arom_, *J* = 7.8 Hz), 6.89 (d, 2H, H_arom_, *J* = 8.8 Hz), 7.17–7.21 (t, 1H, H_arom_, *J* = 7.8 Hz), 7.40-7.43 (m, 3H, H_arom_), 7.79 (s, 1H, CH), 8.65 (s, 2H, OH), 11.97 (s, 1H, NH); MS (EI) *m/z* (%) 475 [M^+^] (95). Anal. Calcd. for C_20_H_17_N_3_O_5_S_3_: C, 50.51; H, 3.60; N, 8.84; O, 16.82; S, 20.23. Found: C, 50.65; H, 3.70; N, 8.71; S, 20.22.

*2-{[5-(3-Allyl-2-hydroxybenzylidene)-4-oxo-2-thioxo-1,3-thiazolidin-3-yl]amino}-N-(4-hydroxy-phenyl)-2-thioxoacetamide* (**46**). Yield 81%; m.p. 153–154 °C; ^1^H-NMR: *δ* 3.26–3.28 (m, 2H, H_alk_), 5.01–5.05 (m, 1H, H_alk_), 5.12–5.18 (m, 1H, H_alk_), 6.02–6.12 (m, 1H, H_alk_), 6.89 (d, 1H, H_arom_, *J* = 9 Hz), 6.99 (d, 1H, H_arom_, *J* = 7.8 Hz), 7.11–7.15 (t, 1H, H_arom_, *J* = 7.8 Hz), 7.42 (d, 2H, H_arom_, *J* = 8.9 Hz), 7.52 (d, 1H, H_arom_, *J* = 7.8 Hz), 7.79 (s, 1H, CH), 7.86 (s, 2H, OH), 10.41 (s, 1H, NH); MS (EI) *m/z* (%) 471 [M^+^] (48). Anal. Calcd. for C_21_H_17_N_3_O_4_S_3_: C, 53.49; H, 3.63; N, 8.91; O, 13.57; S, 20.40. Found: C, 53.55; H, 3.67; N, 8.85; S, 20.43.

*2-({5-[4-(Diethylamino)-2-hydroxybenzylidene]-4-oxo-2-thioxo-1,3-thiazolidin-3-yl}amino)-N-(4-hydroxyphenyl)-2-thioxoacetamide* (**47**). Yield 90%; m.p. 206–207 °C; ^1^H-NMR: δ 1.07–1.12 (t, 6H, CH3, J = 7.2 Hz), 3.24–3.3 (q, 4H, CH2, J = 7.2 Hz), 6.5 (s, 1H, H_arom_), 6.61 (d, 1H, H_arom_, J = 9 Hz), 6.89 (d, 2H, H_arom_, J = 8.9 Hz), 7.32 (d, 1H, H_arom_, J = 9 Hz), 7.43 (d, 2H, H_arom_, J = 8.9 Hz), 7.82 (s, 1H, CH), 8.13 (s, 2H, OH), 10.69 (s, 1H, NH); MS (EI) m/z (%) 502 [M^+^] (84). Anal. Calcd. for C_22_H_22_N_4_O_4_S_3_: C, 52.57; H, 4.41; N, 11.15; O, 12.73; S, 19.14. Found: C, 52.61; H, 4.43; N, 11.22; S, 19.13.

*N-(4-hydroxyphenyl)-2-[(5-{2-hydroxy-5-[(E)-phenyldiazenyl]benzylidene}-4-oxo-2-thioxo-1,3-thiaz-olidin-3-yl)amino]-2-thioxoacetamide* (**48**). Yield 43%; m.p. 145–146 °C; ^1^H-NMR: δ 6.89 (d, 2H, H_arom_, J = 9 Hz), 7.13 (d, 1H, H_arom_, J = 8.5 Hz), 7.34–7.46 (m, 3H, H_arom_), 7.67 (d, 1H, H_arom_, J = 8.5 Hz), 7.84–7.93 (m, 4H, H_arom_), 7.99 (s, 2H, OH), 9.42 (s, 1H, NH); MS (EI) m/z (%) 535 [M^+^] (33). Anal. Calcd. for C_24_H_17_N_5_O_4_S_3_: C, 53.82; H, 3.20; N, 13.08; O, 11.95; S, 17.96. Found: C, 53.91; H, 3.24; N, 13.15; S, 17.92.

*2-Hydroxy-5-{[3-({2-[(4-hydroxyphenyl)amino]-2-oxoethanethioyl}amino)-4-oxo-2-thioxo-1,3-thiazolidin-5-ylidene]methyl}benzoic acid* (**49**). Yield 76%; m.p. 223–224 °C; ^1^H-NMR: *δ* 6.89 (d, 2H, H_arom_, *J* = 9 Hz), 7.2 (d, 1H, H_arom_, *J* = 8.6 Hz), 7.42 (d, 2H, H_arom_, *J* = 9 Hz), 7.59 (s, 1H, CH), 7.82 (d, 1H, H_arom_, *J* = 8.6Hz), 8.17(s, 1H, H_arom_), 9.27 (s, 2H, OH), 10.99 (s, 1H, NH); MS (EI) *m/z* (%) 475 [M^+^] (82). Anal. Calcd. for C_19_H_13_N_3_O_6_S_3_: C, 47.99; H, 2.76; N, 8.84; O, 20.19; S, 20.23. Found: C, 48.05; H, 2.81; N, 8.79; S, 20.22.

*2-{[5-(3,5-Dichloro-2-hydroxybenzylidene)-4-oxo-2-thioxo-1,3-thiazolidin-3-yl]amino}-N-(4-hydroxy-phenyl)-2-thioxoacetamide* (**50**). Yield 53%; m.p. 217–218 °C; ^1^H-NMR: *δ* 6.89 (d, 2H, H_arom_, *J* = 9 Hz), 7.37–7.43 (m, 3H, H_arom_), 7.63 (s, 1H, CH), 7.91 (s, 1H, H_arom_), 9.41 (s, 2H, OH), 11.93 (s, 1H, NH); MS (EI) *m/z* (%) 500 [M^+^] (77). Anal. Calcd. for C_18_H_11_Cl_2_N_3_O_4_S_3_: C, 43.21; H, 2.22; Cl, 14.17; N, 8.40; O, 12.79; S, 19.22. Found: C, 43.18; H, 2.20; Cl, 14.24; N, 8.39; S, 19.19.

*2-{[5-(3,5-Dibromo-2-hydroxybenzylidene)-4-oxo-2-thioxo-1,3-thiazolidin-3-yl]amino}-N-(4-hydroxy-phenyl)-2-thioxoacetamide* (**51**). Yield 87%; m.p. 189–190 °C; ^1^H-NMR: *δ* 6.89 (d, 2H, H_arom_, *J* = 8.8 Hz), 7.43 (d, 2H, H_arom_, *J* = 8.8 Hz), 7.59 (s, 1H, H_arom_), 7.73 (s, 2H, CH, H_arom_), 8.93 (s, 2H, OH), 11.02 (s, 1H, NH); MS (EI) *m/z* (%) 589 [M^+^] (70). Anal. Calcd. for C_18_H_11_Br_2_N_3_O_4_S_3_: C, 36.69; H, 1.88; Br, 27.12; N, 7.13; O, 10.86; S, 16.32. Found: C, 36.68; H, 1.82; Br, 27.17; N, 7.10; S, 16.29.

*2-{[5-(5-Bromo-2-hydroxy-3-methoxybenzylidene)-4-oxo-2-thioxo-1,3-thiazolidin-3-yl]amino}-N-(4-hydroxyphenyl)-2-thioxoacetamide* (**52**). Yield 74%; m.p. 176–177 °C; ^1^H-NMR: *δ* 3.76 (s, 3H, OCH_3_), 6.89 (m, 3H, H_arom_), 7.42 (m, 3H, H_arom_), 7.74 (s, 1H, CH), 8.65 (s, 2H, OH), 10.12 (s, 1H, NH); MS (EI) *m/z* (%) 540 [M^+^] (91). Anal. Calcd. for C_19_H_14_BrN_3_O_5_S_3_: C, 42.23; H, 2.61; Br, 14.79; N, 7.78; O, 14.80; S, 17.80. Found: C, 42.29; H, 2.66; Br, 14.82; N, 7.74; S, 17.76.

*2-{[5-(2-Hydroxy-3,5-diiodobenzylidene)-4-oxo-2-thioxo-1,3-thiazolidin-3-yl]amino}-N-(4-hydroxy-phenyl)-2-thioxoacetamide* (**53**). Yield 56%; m.p. 157–158 °C; ^1^H-NMR: *δ* 6.89 (d, 2H, H_arom_, *J* = 8.9 Hz), 7.42 (m, 3H, H_arom_), 7.63 (s, 1H, CH), 7.83 (s, 1H, H_arom_), 8.94 (s, 2H, OH), 10.86 (s, 1H, NH); MS (EI) *m/z* (%) 683 [M^+^] (15). Anal. Calcd. for C_18_H_11_I_2_N_3_O_4_S_3_: C, 31.64; H, 1.62; I, 37.14; N, 6.15; O, 9.37; S, 14.08. Found: C, 31.60; H, 1.58; N, 6.19; S, 14.05.

*2-{[5-(2-Hydroxy-3,5-diisopropylbenzylidene)-4-oxo-2-thioxo-1,3-thiazolidin-3-yl]amino}-N-(4-hydroxyphenyl)-2-thioxoacetamide* (**54**). Yield 77%; m.p. 184–185 °C; ^1^H-NMR: *δ* 1.1–1.15 (m, 12H, H_alk_), 2.45–2.54 (m, 1H, H_alk_), 3.14–3.21 (m, 1H, H_alk_), 6.8 (s, 1H, H_arom_), 6.89 (d, 2H, H_arom_, *J* = 8.9 Hz), 7.16 (s, 1H, H_arom_), 7.42 (d, 2H, H_arom_, *J* = 8.8 Hz), 7.67 (s, 1H, CH), 7.85 (s, 2H, OH), 10.13 (s, 1H, NH); MS (EI) *m/z* (%) 515 [M^+^] (83). Anal. Calcd. for C_24_H_25_N_3_O_4_S_3_: C, 55.90; H, 4.89; N, 8.15; O, 12.41; S, 18.65. Found: C, 55.97; H, 4.94; N, 8.10; S, 18.62.

### 3.3. In Vitro Enzyme Inhibition Assays

#### 3.3.1. Materials, Chemicals and Enzymes

All compounds were dissolved in DMSO to a final stock concentration of 10 mM and stored at −20 °C. Initial screening for IN inhibitory effect was done at 100 μM and dilutions were made in DMSO. Compounds showing at least 50% inhibition at 100 μM were selected for IC_50_ determinations. γ-[^32^P]-ATP was purchased from Perkin Elmer (Waltham, MA, USA). The expression system for wild-type IN was a generous gift of Dr. Robert Craigie, Laboratory of Molecular Biology, NIDDK, NIH (Bethesda, MD, USA). His-tagged APE1 was purified from an expression system kindly provided by Dr. Tom Curran, Department of Developmental Neurobiology, St. Jude Children’s Research Hospital (Memphis, TN, USA).

#### 3.3.2. Preparation of Oligonucleotide Substrates

21-mer oligonucleotides used in IN inhibition assay [21top (5’-GTGTGGAAAATCTCTAGCAGT-3’) and 21bot (5’-ACTGCTAGAGATTTTCCA CAC-3’)] were purchased from Integrated DNA Technologies (Coralville, IA, USA) and purified by UV shadowing on polyacrylamide gel. The oligonucleotides used in APE1 inhibition assay [top strand: 5′-ATTTCACCGGTACG(F)TCTAGAATC CG-3′ containing the tetrahydrofuran synthetic abasic residue (F), and bottom strand: 3′-TAAAGTGG CCATGC(C)AGATCTTAGGC-5′] were purchased from The Midland Certified Reagent Company (Midland, TX, USA). To analyze the extent of 3’-processing and strand transfer with 5’-end labeled substrates, 21top was 5’-end labeled using T4 polynucleotide kinase (Epicentre, Madison, WI, USA) and [γ-^32^P]-ATP (Perkin Elmer, Waltham, MA, USA). The kinase was heat-inactivated at 95 °C and 21bot was added in 1.5 M excess. The reaction mixture was allowed to cool slowly to room temperature, and purified through a spin-25 minicolumn (USA Scientific, Ocala, FL, USA) to separate annealed double-stranded oligonucleotide from unincorporated material. The oligonucleotides used in the APE1 inhibition assay were labeled using a similar protocol. 

#### 3.3.3. *In Vitro* IN Inhibition Assay

To determine the extent of 3’-processing and strand transfer, the test compounds, diluted in DMSO, were pre-incubated with purified recombinant IN (at a final concentration of 200 nM) in the reaction buffer [50 mM NaCl, 1 mM HEPES (pH 7.5), 50 μM EDTA, 50 μM dithiothreitol, 10% glycerol (w/v), 7.5 mM MnCl2, 0.1 mg mL–1 bovine serum albumin, 10 mM 2-mercaptoethanol, 10% dimethyl sulfoxide, and 25 mM MOPS (pH 7.2)] at 30 °C for 30 min. For Mg^2+^ assay, 7.5 mM MgCl_2_ was substituted for MnCl_2_ and an optimal concentration of PEG was included in the reaction buffer. Following the pre-incubation, 20 nM of the 5’-end ^32^P-labeled linear oligonucleotide substrate was added to the reaction mixture and incubated for an additional 1 h. Reactions were quenched by addition of 50X loading dye (98% deionized formamide, 10 mM EDTA, 0.025% xylene cyanol, and 0.025 % bromophenol blue). An aliquot was subjected to electrophoresis on a denaturing polyacrylamide gel (0.09 M tris–borate, pH 8.3, 2 mM EDTA, 20% acrylamide, 8M urea). Gels were dried under vacuum, exposed in a PhosphorImager cassette and visualized with a Typhoon 8610 Variable Mode Imager (Amersham Biosciences). Quantification was done with Image Quant 5.2 software. Percent inhibition (%I) was calculated using the following equation:

%I = 100 × [1 − (D − C) / (N − C)]

where C, N, and D are the fractions of 21-mer substrate converted into 19-mer (product of 3’-processing) or strand-transfer products for DNA alone, DNA plus IN, and DNA, IN plus test compound, respectively. IC_50_ values were determined by plotting the logarithm of test compound concentrations as a function of %I to obtain the concentration that produced 50% inhibition.

#### 3.3.4. APE1 Inhibition Assay

APE1 inhibition assay was performed as previously described [26]. To determine the extent of endonucleolytic cleavage of the abasic residue, the test compounds, diluted in DMSO, were preincubated with recombinant APE1 at a final concentration of 0.05 nM in the reaction buffer [50 mM NaCl, 1 mM HEPES, pH 7.5, 50 μM EDTA, 50 μM dithiothreitol, 10% glycerol (w/v), 7.5 mM MnCl2, 0.1 mg mL‑1 bovine serum albumin, 10 mM 2-mercaptoethanol, 10% DMSO, and 25 mM MOPS, pH 7.2] at 30 °C for 10 min. Following preincubation, 200 nM of the 5′-end ^32^P-labeled linear oligonucleotide substrate was added, and incubated for an additional 10 minutes. Reactions were quenched by the addition of an equal volume (16 μL) of loading dye (98% deionized formamide, 10 mM EDTA, 0.025% xylene cyanol, and 0.025% bromophenol blue). An aliquot (5 μL) was electrophoresed on a denaturing 20% polyacrylamide gel (0.09 M tris-borate pH 8.3, 2 mM EDTA, 20% acrylamide, 8 M urea). The gels were dried, exposed in a PhosphorImager cassette and analyzed using a Typhoon 8610 Variable Mode Imager (Amersham Biosciences). Quantification of enzyme inhibition was done using Image Quant 5.2 software. The percent inhibition (% I) was calculated using the following equation:

%I = 100 × [1 − (D − C) / (N − C)]

where C, N, and D are the fractions of 26-mer substrate converted to 13-mer incision products (or integration products) for DNA alone, DNA plus APE1, and DNA, APE1 plus test compound, respectively. IC_50_ values were determined by plotting the logarithm of compound concentration as a function of %I to obtain the concentration that produced 50% inhibition. An optimum concentration of APE1 enzyme, at which there is complete conversion of the synthetic abasic-site containing oligonucleotide substrate to its cleaved product without subsequent exonuclease activity, was used in the assay.

### 3.4. Docking Studies

#### 3.4.1. GOLD

*HIV-1 IN*. Structures of all the compounds **6**–**54** from Table 1–Table 5 were built and energy minimized using Catalyst (Accelrys, Inc., San Diego, CA, USA) [35]. Using the Poling algorithm and the best flexible conformation generation method in Catalyst, feasible and unique conformations for each compound was generated over a 20 kcal/mol range of energies. Three-dimensional coordinates of the crystallized structure of the catalytic domain of HIV-1 IN were obtained from the Protein Data Bank at the Research Collaboratory for Structural Bioinformatics (RCSB PDB; PDB ID: 1BL3). All the water molecules present in protein were removed. Hydrogen atoms were added to the protein to assign appropriate ionization states to both the acidic and basic amino acid residues in the active site. Docking was performed using version 4.0 of the GOLD (Genetic Optimization for Ligand Docking; Cambridge Crystallographic Data Centre) software package [36]. A 20 Å radius, centered at the carboxylate oxygen atom (OD1) of the amino acid residue D64, within the IN active site was defined as the binding site. The docking simulations were carried out using the standard default settings—a population size of 100, a maximum number of 100,000 operations, and a mutation and crossover rate of 95. The GOLD Score fitness function is the default scoring function provided with GOLD. It has been optimized for the prediction of ligand binding positions and takes into account factors like H-bonding energy, Van der Waals energy and ligand torsion strain. On the other hand, the ChemScore fitness function estimates the total free energy change that occurs on ligand binding and is trained by regression against binding affinity data. All the compounds (**6**–**54**) were docked into the defined IN active site and the GOLD fitness scores were calculated. For each molecule, the bound conformation with a high fitness score was considered as the best bound conformation.

*APE1*. Three-dimensional coordinates of the crystallized structure of human APE1 endonuclease with bound abasic DNA and Mn^2+^ ion were obtained from RCSB PDB (PDB ID: 1DE9). For the protein target, a 20 Å radius active site was defined using xyz coordinates of the 5’-phosphate fragment of the abasic DNA in the co-crystal structure. Prior to docking, the bound abasic DNA and chain B of the APE1 monomer were removed, and proper protonation states were assigned for the acidic and basic residues. Docking simulations with standard parameters and GOLD fitness score calculations were performed as described above. Based on the GOLD fitness scores for each molecule, a bound conformation with high fitness score was considered as the best bound-conformation. 

#### 3.4.2. Glide

Compounds **6–54** from Table 1-Table 5 were processed using the Schrödinger LigPrep utility (Schrödinger, LLC, USA). This program produces a number of low energy 3D structures, with various ionization states, tautomers, stereochemistries, and ring conformations, from each molecule input. For this study, a pH range of 6-8 was used. Both the neutral and the anionic states of carboxylic acid groups were generated. Three-dimensional coordinates of the crystal structures of HIV-1 IN catalytic domain and human APE1 endonuclease, with bound abasic DNA and Mn^2+^ ion, were obtained from the RCSB PDB (PDB ID: 1BL3 and 1DE9). All the water molecules present in the protein were removed, and hydrogen atoms were added considering the appropriate ionization states for both the acidic and basic amino acid residues. All compounds (**6–54**) were docked onto the binding site in HIV-1 IN or human APE1 protein using version 5.0 of Glide (Grid-Based Ligand Docking With Energetics) software from Schrödinger [37,38,39]. A grid with a bounding box size of 20 Å was used. The coordinates of the enclosing box (1BL3: x = 22.23 Å; y = 1.33 Å; z = −19.16 Å and 1DE9: x = 47.866; y = 9.534; z = 40.274) were defined starting from the set of active site residues involved. The Glide algorithm is based on a systematic search of positions, orientations, and conformations of the ligand in the receptor binding site using funnel type approach. The search begins with a rough positioning and scoring phase. This significantly limits the search space, and reduces the number of poses to be selected for minimization on the precomputed OPLS-2005 Van der Waals and electrostatic grids for the protein. In order to obtain an accurate correlation between good poses and good scores, the Glide Extra-Precision (XP) Mode was used.

### 3.5. Drug-Like Property Prediction

The ligand structures were energy minimized using the Catalyst software package (Accelrys, Inc.) [35] and the lowest- energy conformation of each compound was exported to ADMET Predictor (Simulations Plus, Inc., Lancaster, CA, USA) [40] to calculate various ADME (absorption, distribution, metabolism and excretion) properties.

### 3.6. Biological Activity

#### 3.6.1. Cell Lines and Chemicals

Human melanoma cell line MDA-MB 435, pancreatic cancer cell line Panc-1, and colon cancer cell line HT 29 were purchased from the American Type Cell Culture (Manassas, VA, USA). HCT 116 p53^+/+^ and HCT 116 p53^−/^^−^ were kindly provided by Dr. Bert Vogelstein, Johns Hopkins Medical Institutions (Baltimore, MD, USA). HCT 116 p53^+/+^, HCT116 p53^−/^^−^, and HT 29 cells were maintained as monolayer cultures in RPMI 1640 supplemented with 10% fetal bovine serum (FBS; Gemini-Bioproducts, Woodland, CA, USA) and 2 mmol/L L-glutamine at 37 °C in a humidified atmosphere of 5% CO_2_. Similarly, MDA-MB 435 and Panc-1 cells were maintained in Dulbecco’s modified Eagle’s medium (DMEM) supplemented with 10% fetal bovine serum as described above. To remove the adherent cells from the flask for passaging and counting, cells were washed with PBS without calcium or magnesium, incubated with a small volume of 0.25% trypsin-EDTA solution (Sigma-Aldrich, St. Louis, MO, USA) for 5 to 10 minutes and washed with culture medium and centrifuged. All experiments were done using cells in exponential cell growth.

#### 3.6.2. Cytotoxicity Assay

Cytotoxicity was assessed by 3-(4,5-dimethylthiazol-2-yl)-2,5-diphenyltetrazolium bromide (MTT) assay as previously described [41,42]. Briefly, cells were seeded in 96-well microtitre plates (Panc-1 and HT 29 cells were seeded at 8,000 cells/well, and MDA-MB-435 and HCT116 cells at 4,000 cells/well, respectively). After overnight attachment, cells were treated with the test compounds for 72 h. Initial screening in MTT assays was performed at 10 μM using sterile drug solutions prepared in suitable cell culture media (final DMSO concentration in drug dilutions was less 0.001%). Compounds showing at least 50% growth inhibition at 10 μM were selected for IC_50_ determinations. 

After 72 h exposure to the test compounds, MTT solution (5 mg/mL; 20 μL) was added to each well and cells were incubated for 4 h at 37 °C. After incubation, media from each well was removed and the dark blue formazan crystals formed by live cells were dissolved in DMSO (150 μL/well). The absorbance intensity was measured at 570 nm against appropriate blank controls using a microplate reader (Molecular Devices, Sunnyvale, CA, USA). All assays were done in triplicate. The growth inhibitory concentration GI_50_ was determined by plotting the logarithm of compound concentration against percentage of cells killed to obtain the concentration that produced 50% cell kill.

## 4. Conclusions

In this study, we have investigated the SAR of rhodanine-based IN inhibitors by modifying the aryl and alkylidene sub-groups. Several of these compounds showed moderate to potent IN inhibition and appeared to be more selective for IN as compared to APE1. Results from this study confirm the importance of the rhodanine moiety as a useful structural scaffold with promising IN inhibitory activity and provides a framework for designing more potent and selective IN inhibitors. In addition, some of these compounds inhibited APE1 endonuclease activity and also showed antiproliferative effects against cancer cell lines; thus making rhodanine-based derivatives suitable leads for antiviral and anticancer drug development. 
